# Biased activation of β_2_-AR/Gi/GRK2 signal pathway attenuated β_1_-AR sustained activation induced by β_1_-adrenergic receptor autoantibody

**DOI:** 10.1038/s41420-021-00735-2

**Published:** 2021-11-08

**Authors:** Hao Chen, Ning Cao, Li Wang, Ye Wu, Haojie Wei, Yuming Li, Youyi Zhang, Suli Zhang, Huirong Liu

**Affiliations:** 1 Department of Physiology & Pathophysiology, School of Basic Medical Sciences, Capital Medical University, Beijing, 100069 PR China; 2 Institute of Vascular Medicine, Cardiology Department, Peking University Third Hospital, Beijing, 100191 PR China; 3 Department of Pathology, School of Basic Medical Sciences, Shanxi Medical University, Taiyuan, 030001 PR China; 4 Department of Physiology & Pathophysiology, Yanjing Medical College, Capital Medical University, Beijing, 101300 PR China; 5 Beijing Key Laboratory of Metabolic Disorders Related Cardiovascular Disease, Capital Medical University, Beijing, 100069 PR China

**Keywords:** Heart failure, Hormone receptors

## Abstract

Heart failure is the terminal stage of many cardiac diseases, in which β_1_-adrenoceptor (β_1_-AR) autoantibody (β_1_-AA) has a causative role. By continuously activating β_1_-AR, β_1_-AA can induce cytotoxicity, leading to cardiomyocyte apoptosis and heart dysfunction. However, the mechanism underlying the persistent activation of β_1_-AR by β_1_-AA is not fully understood. Receptor endocytosis has a critical role in terminating signals over time. β_2_-adrenoceptor (β_2_-AR) is involved in the regulation of β_1_-AR signaling. This research aimed to clarify the mechanism of the β_1_-AA-induced sustained activation of β_1_-AR and explore the role of the β_2_-AR/Gi-signaling pathway in this process. The beating frequency of neonatal rat cardiomyocytes, cyclic adenosine monophosphate content, and intracellular Ca^2+^ levels were examined to detect the activation of β_1_-AA. Total internal reflection fluorescence microscopy was used to detect the endocytosis of β_1_-AR. ICI118551 was used to assess β_2_-AR/Gi function in β_1_-AR sustained activation induced by β_1_-AA in vitro and in vivo. Monoclonal β_1_-AA derived from a mouse hybridoma could continuously activate β_1_-AR. β_1_-AA-restricted β_1_-AR endocytosis, which was reversed by overexpressing the endocytosis scaffold protein β-arrestin1/2, resulting in the cessation of β_1_-AR signaling. β_2_-AR could promote β_1_-AR endocytosis, as demonstrated by overexpressing/interfering with β_2_-AR in HL-1 cells, whereas β_1_-AA inhibited the binding of β_2_-AR to β_1_-AR, as determined by surface plasmon resonance. ICI118551 biasedly activated the β_2_-AR/Gi/G protein-coupled receptor kinase 2 (GRK2) pathway, leading to the arrest of limited endocytosis and continuous activation of β_1_-AR by β_1_-AA in vitro. In vivo, ICI118551 treatment attenuated myocardial fiber rupture and left ventricular dysfunction in β_1_-AA-positive mice. This study showed that β_1_-AA continuously activated β_1_-AR by inhibiting receptor endocytosis. Biased activation of the β_2_-AR/Gi/GRK2 signaling pathway could promote β_1_-AR endocytosis restricted by β_1_-AA, terminate signal transduction, and alleviate heart damage.

## Introduction

Heart failure (HF) is the result of heart diseases and is a global threat to human health. Overactivation of the β_1_-adrenergic receptor (β_1_-AR) in cardiomyocytes is a core HF mechanism [1]. We and others have shown that in addition to the endogenous β_1_-AR ligand norepinephrine (NE), autoantibodies against β_1_-AR (β_1_-AA), harboring agonist-like effects, circulate in the sera of 30–40% of HF patients [2, 3]. Studies have demonstrated that β_1_-AA can directly lead to HF [4], suggesting the importance of β_1_-AA in HF development.

There are several potential approaches to protect against cardiac damage caused by β_1_-AA. β-blockers are widely used in treating chronic HF by blocking β_1_-AR activation [5]. However, the β_1_-AA-included pathological effects can only be partially abrogated by β-blockers, as suggested by animal [6] and clinical studies [7]. In vitro plasma exchange or immunoadsorption methods can increase the cardiac ejection fraction and restore cardiac function to a certain extent. However, these two schemes have poor specificity. Other approaches, such as peptide homologs mimicking the epitope(s) or aptamers, are still underway. One of the reasons for the shortcomings of treatment plans is our insufficient understanding of the characteristics of β_1_-AA-mediated activation of β_1_-AR.

In contrast to transiently activating β_1_-AR by NE, β_1_-AA can persistently activate β_1_-AR, which leads to the overactivation of β_1_-AR downstream signaling and cardiac injury. Existing studies have suggested that this mode of action of β_1_-AA may be related to decreased β_1_-AR endocytosis [8]. However, there is a lack of convincing evidence on the role of limited endocytosis in the sustained activation of β_1_-AR caused by β_1_-AA and the related mechanisms. β_2_-adrenergic receptor (β_2_-AR) is another important adrenergic receptor in cardiomyocytes [9]. Our previous studies unexpectedly found that although β_1_-AA does not directly bind to β_2_-AR, the sustained increase in NRCM-beating frequency induced by β_1_-AA can be neutralized by the β_2_-AR autoantibody-mediated activation of β_2_-AR [2], indicating cross-talk between β_2_-AR and β_1_-AR signals under the action of β_1_-AA. Similar to β_1_-AR, β_2_-AR can couple to Gs protein and stimulate adenylyl cyclase (AC) [9]. The difference is that β_2_-AR can also couple to Gi protein, which mediates an opposite effect [9]. For example, upregulated β_2_-AR expression negatively regulates the overcontraction of cardiomyocytes caused by β_1_-AR through the Gi-signaling pathway in HF [10]. Utilizing a hypothesis-driven approach, we speculated that the insufficient regulatory function of the β_2_-AR/Gi pathway might be involved in β_1_-AA-induced limited β_1_-AR endocytosis and persistent signaling. Strengthening the β_2_-AR/Gi pathway may be a new rescue strategy for β_1_-AA-positive HF.

## Materials and methods

### Animals

The animals used in this study were 6- to 8-week-old Balb/c mice and 0- to 3-day-old neonatal rats. The animals were fed in the SPF animal room of Capital Medical University. At the end of the experiment, the mice were euthanized with an intraperitoneal injection of 40 mg/kg sodium pentobarbital.

### Patients

All patients gave informed consent. The detailed screening process is provided in the supplemental material. The information of patients was shown in Table S1.

### Ethics statement

All animal experiments in this study complied with the National Institutes of Health Guide for the Care and Use of Laboratory Animals. This study was approved by the Institutional Animal Care and Use Committee of Capital Medical University. The study complied with the journal’s applicable checklists for animal ethics (AEEI-2015-097, AEEI 2016-053).

The research study in patients complied with the Declaration of Helsinki and passed the approval of the Beijing Anzhen Hospital’s Ethical Review Committee.

### NRCMs beating frequency and cyclic adenosine monophosphate measurements

Neonatal rats (0- to 3-day-old) were provided by the Capital Medical University animal laboratory. Neonatal rats were sacrificed by carbon dioxide inhalation. Hearts were immediately collected in iced-cold PBS. Ventricular cardiomyocytes were extracted by an enzymatic method and cultured in a cell incubator [11]. The spontaneous NRCMs contraction rate was monitored with a living cell station [2].

The cAMP content in NRCMs was measured by a cAMP [^125^I] radioimmunoassay (RIA) kit (RK-509, Hungary) [12]. The detailed experimental procedure was provided in the supplemental material.

### Total internal reflection fluorescence microscopy

Total internal reflection fluorescence microscopy (TIRF) was utilized to determine β_1_-AR endocytosis in HL-1 cells transfected with β_1_-AR-GFP. The myocardial cell line HL-1 was cultured in confocal dishes. A TIRF inverted microscope (Olympus, Japan) equipped with an EMCCD camera and an oil immersion objective lens (×100 magnification, NA = 1.49) was utilized. The depth of field, 120-130 nm, was selected to observe β_1_-AR fluorescence upon the plasma membrane but not the cytoplasm. Changes in cell surface fluorescence were recorded at 0, 5, 10, and 30 minutes (min) after cell administration. Recordings were made every 30 seconds (s) using MetaMorph software version 7.8.8.0. Image fluorescence intensity was analyzed using ImageJ 1.51J8 [13]. To detect the recruitment of β_1_-AR-GFP and β-arrestin-RFP, we recorded images at 5 min after drug treatment and utilized ImageJ 1.53c for colocalization analysis. Before analysis, all images were adjusted with ImageJ 1.53c to the same brightness and color. Pearson’s *R* value (above threshold) was used as an indicator of the colocalization of β_1_-AR-GFP and β-arrestin-RFP [14].

### Intracellular calcium ion detection

HL-1 cells were cultured at a density of ~30% in confocal dishes. The cells were incubated with the Ca^2+^ fluorescent probe Fluo-4 (10 μM, F14201, Thermo Fisher, USA) for 90 min before the experiment. At the time of the experiment, the cell culture medium was changed to FluoroBrite DMEM (A1896701, Thermo Fisher, USA). Intracellular Ca^2+^ levels were measured using a turntable confocal microscope (UltraVIEW VoX, USA) [13].

### Antibodies

Please refer to supplementary table S2 for detailed information.

### Establishment of a mouse heart dysfunction model by injecting β_1_-AA

A mouse monoclonal β_1_-AA (synthesized from AbMax Biotechnology, China) [15] was mixed with saline before use (concentration of 0.5 mg/ml). Heart dysfunction models were established in male 6 to 8-week-old Balb/c mice randomly by intraperitoneal β_1_-AA injection (10 μg/kg, every two weeks, i.p.). In β_2_-AR inverse agonist treatment group, 32.7 μg/kg ICI118551 was injected intraperitoneally every week for 2 months, accompanied by β_1_-AA injection every two weeks. Left ventricular function was determined in all mice by echocardiography, and morphological changes in the heart were observed by HE staining.

### Statistical analysis

In this study, GraphPad Prism 8.0.2 software was used for statistical analysis and statistical graph production. The experimental results are expressed as the mean ± SEM. The differences in dynamic changes in NRCM-beating frequency, cAMP concentration, and β_1_-AR endocytosis in the different groups were analyzed by 2-way ANOVA followed by a Bonferroni post hoc test. One-way ANOVA followed by Dunnett’s test was used to compare multiple groups, and the t-test was used to compare the mean between two groups. *P* < 0.05 was considered statistically significant.

The detail of other material and methods was described in the supplementary material.

## Results

### Similar to β_1_-AA (+)-IgG from patients, mouse-derived monoclonal β_1_-AA induces sustained activation and limited endocytosis of β_1_-AR

**Fig. 1:**
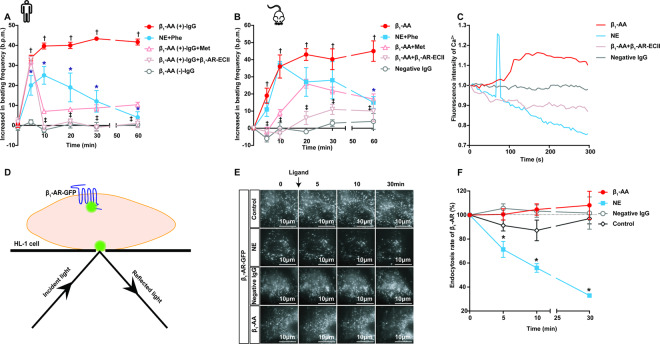
Monoclonal β_1_-AA purified from mouse-derived hybridoma cells caused long-term activation of β_1_-AR but restricted β_1_-AR endocytosis. **A** and **B** NRCMs were utilized to evaluate the effects of β_1_-AA (+)-IgG (β_1_-AA-positive total IgG) isolated from HF patients and mouse-derived monoclonal β_1_-AA on β_1_-AR activation. β_1_-AA (-)-IgG (β_1_-AA-negative total IgG) from HF patients and negative IgG from healthy mice were treated as the antibody controls. NE acted as a physiological ligand control. Phe and Met were α-adrenoceptor (α-AR) and β_1_-AR blockers, respectively. The peptide-based on β_1_-AR-ECII could specifically neutralize β_1_-AA. Data were analyzed by two-way ANOVA, followed by a Bonferroni post hoc test. *n* = 4–6/group, *P vs β_1_-AA, ^†^*P* vs β_1_-AA (-)-IgG (**A**) or negative IgG (**B**), ^‡^*P* vs β_1_-AR-ECII, *P* < 0.05. **C** HL-1 cells were incubated with Fluo-4 (10 μM) for 90 min and then treated with different stimuli to dynamically measure the intracellular Ca^2+^ changes. *n* = 3/group. **D**–**F** TIRF was used to determine the endocytosis of β_1_-AR on the membrane of HL-1 cells, reflected by the decreased percentage of fluorescence value of β_1_-AR-GFP. **D** is the schematic diagram of TIRF detection. **E** and **F** are typical pictures and statistical charts of the effects of different stimuli on β_1_-AR-GFP endocytosis, respectively. Scale bar = 10 μm. Data were analyzed by two-way ANOVA, followed by a Bonferroni test. *n* = 3/group. *P vs β_1_-AA, *P* < 0.05.

β_1_-AA and negative IgG used in the current study originated from mouse-derived hybridoma cells and sera from healthy Balb/c mice, respectively. We first compared the effect of mouse-derived β_1_-AA with that from HF patients. As shown in Fig. 1, patients’ β_1_-AA (+)-IgG (β_1_-AA-positive total IgG, 10^−7^ M) elicited persistent activation of β_1_-AR, evidenced by the increasing NRCM-beating frequency for 1 hour, an effect partially inhibited by the β_1_-AR blocker metoprolol (Met, 10^−5^ M) and completely neutralized by the peptide corresponding to the extracellular second loop of β_1_-AR (β_1_-AR-ECII, 10^−5^ M) (Fig. 1A, Table S3). After preincubating NRCMs with phenoxybenzamine (Phe, 10^−5^ M) for 30 min to block the α_1_-adrenergic receptor (α_1_-AR), NE (10^−5^ M) increased the NRCM-beating frequency for a much shorter time, ~5 min (Fig. 1A). The mouse monoclonal β_1_-AA (10^−7^ M) also continuously increased NRCM-beating frequency through β_1_-AR, whereas negative IgG had no effect (Fig. 1B, Table S4). Moreover, monoclonal β_1_-AA increased the Ca^2+^ level in HL-1 cells, which lasted until the end of the observation (300 s). This effect could be blocked by the β_1_-AR-ECII peptide. In contrast, after NE stimulation, the intracellular Ca^2+^ increased rapidly and then decreased rapidly, maintaining for only 5 s (Fig. 1C, Table S5, Video S1, and S2). Met, Phe, and the β_1_-AR-ECII peptide alone had no effects on either NRCM-beating or intracellular Ca^2+^ levels (Fig. S1A, B, Table S7 and S8). These results indicated that the mouse monoclonal β_1_-AA and the β_1_-AA from HF patients acted very similarly, and the former was used in subsequent experiments.

G protein-coupled receptor (GPCR) endocytosis can terminate the downstream signal over time. To investigate the potential role of endocytosis in long-term β_1_-AR activation, we transiently transfected the β_1_-AR-GFP plasmid into an HL-1 cell line (Fig. S2A, B, Fig. S14 A1, A2). TIRF was used to determine the degree of β_1_-AR endocytosis reflected by the reduced β_1_-AR-GFP fluorescence intensity on the cell membrane. TIRF detection showed that the fluorescence value was reduced by 28% at 5 min after treatment with NE plus Phe and by 44% and 67% after 10 min and 30 min (Fig. 1D–F, Fig. S20), respectively, indicating marked β_1_-AR endocytosis. However, the fluorescence intensity showed no significant change in the β_1_-AA treatment group or in the plasmid transfection control and negative IgG groups (Fig. 1F, Table S6).

### Weakened β_1_-AR endocytosis is an important factor in the continuous activation of β_1_-AR

**Fig. 2:**
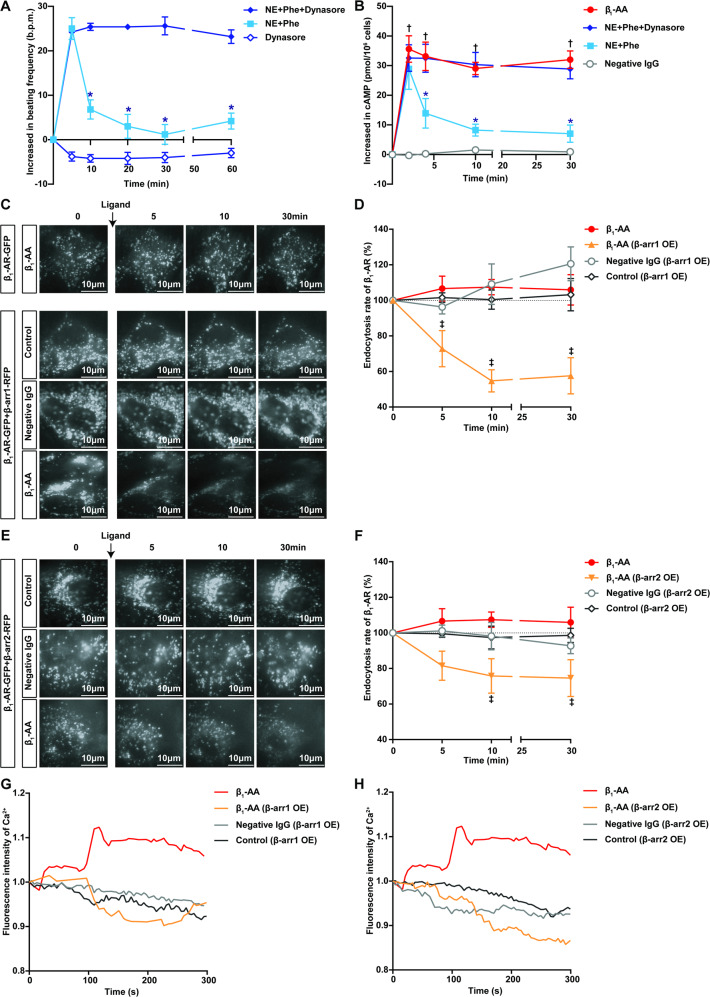
Inhibiting/promoting β_1_-AR endocytosis enhanced/weakened the sustained activation of β_1_-AR. **A** The effect of the receptor endocytosis inhibitor Dynasore on the NE-induced increase in NRCMs beating frequency. *n* = 5/group, *P vs NE + Phe, *P* < 0.05. **B** An radioimmunoassay kit was used to detect the cAMP concentration in NRCMs after treatment with different stimuli. *n* = 4/group. *P vs NE + Phe, ^†^β_1_-AA vs negative IgG, *P* < 0.05. **C**–**F** After overexpressing β-arrestin1 (β-arr1 OE) or β-arrestin2 (β-arr2 OE), the effect of β_1_-AA on β_1_-AR-GFP endocytosis was measured via TIRF. HL-1 cells were transiently transfected with β_1_-AR-GFP, combined with or without transfection with β-arrestin1-RFP (**C** and **D**) or β-arrestin2-RFP (**E** and **F**). **C** and **E** are typical pictures, and **D** and **F** are the corresponding statistical charts. Scale bar = 10 μm. *n* = 3–4/group. ^‡^*P* vs β_1_-AA, *P* < 0.05. Data in **A**–**F** were analyzed by two-way ANOVA with a Bonferroni post hoc test. **G** and **H** The impacts of β_1_-AA on intracellular Ca^2+^ in HL-1 cells with or without β-arr1 OE or β-arr2 OE. Fluo-4 (10 μM) was used as a Ca^2+^ indicator. *n* = 3–4/group.

To clarify the causal relationship between endocytosis and β_1_-AR activation duration, we assessed the changes in β_1_-AR-mediated signaling and the effect after decreasing or increasing β_1_-AR endocytosis. As shown in Fig. 2A, after blocking α_1_-AR with Phe (10^−5^ M), NE (10^−5^ M) induced a short-term increase in NRCMs beating and intracellular cyclic adenosine monophosphate (cAMP) levels. In contrast, after preincubation with the GPCR endocytosis inhibitor Dynasore (10^−4^ M) for 30 min, NE increased the beating frequency of NRCMs for 60 min and the cAMP level for 30 min, similar to that caused by β_1_-AA (Fig. 2A, B, Tables S9 and S10). The result of Dynasore treatment alone was negative for the NRCMs beating (Fig. 2A).

β-arrestin1/2 are key scaffold proteins that initiate β_1_-AR endocytosis. Bioluminescence resonance energy transfer detection showed that it was difficult for β_1_-AR to recruit β-arrestin1 or β-arrestin2 upon β_1_-AA stimulation (Fig. S3 A-G, Fig. S14 B1-D2, Tables S15 and S16). Therefore, we overexpressed β-arrestin1-RFP (β-arr1 OE) or β-arrestin2-RFP (β-arr2 OE) in HL-1 cells transiently transfected with β_1_-AR-GFP to improve β_1_-AR endocytosis (Fig. S4 A, B, Fig. S15 A1-C2). As determined by TIRF, either β-arrestin1-RFP or β-arrestin2-RFP overexpression markedly enhanced β_1_-AA-induced β_1_-AR endocytosis. At 30 min after β_1_-AA administration (10^−7^ M), β_1_-AR-GFP fluorescence decreased by 42% in the β-arr1 OE group (Fig. 2C, D, Fig. S21 A, Table S11) and by 25% in the β-arr2 OE group (Fig. 2E, F, Fig. S21 B, Table S12). Moreover, both β-arrestin1-RFP and β-arrestin2-RFP overexpression significantly attenuated the sustained elevated levels of Ca^2+^ in HL-1 cells caused by β_1_-AA (Fig. 2G, H, Tables S13 and S14), suggesting that the prevention of enhanced β_1_-AR endocytosis led to the continuous activation of β_1_-AR. We concluded that β_1_-AA-initiated sustained activation of β_1_-AR could be at least partially attributed to insufficient β_1_-AR endocytosis.

### Insufficient regulation of β_2_-AR is involved in the limited endocytosis and sustained activation of β_1_-AR caused by β_1_-AA

**Fig. 3:**
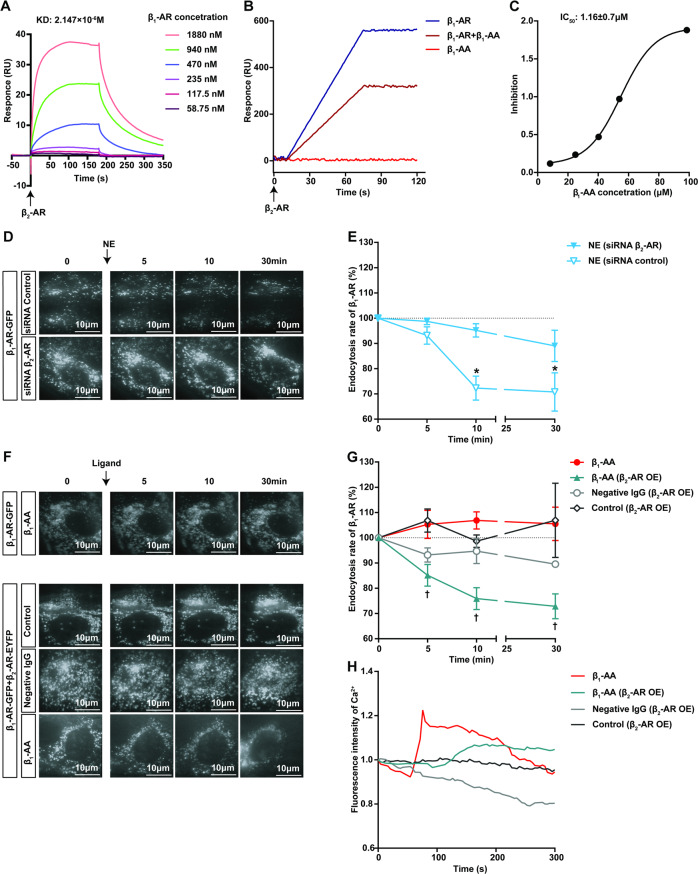
The ability of β_2_-AR to promote β_1_-AR endocytosis was reduced upon β_1_-AA stimulation. **A** The binding ability of purified β_1_-AR to β_2_-AR proteins was detected by SPR. The β_2_-AR protein was first coupled to the chip, and then different concentrations of β_1_-AR proteins were added. **B** SPR was used to observe the binding of β_1_-AA and β_2_-AR protein and the influence of β_1_-AA on the binding of β_1_-AR and β_2_-AR. **C** The IC_50_ of β_1_-AA on binding β_1_-AR and β_2_-AR. **D**, **E** TIRF was used to assess NE-induced β_1_-AR endocytosis when HL-1 cells were transfected with siRNA β_2_-AR or an siRNA control. Representative images and quantification of changes in β_1_-AR-GFP fluorescence density are shown in **D** and **E**, respectively. Scale bar = 10 μm. *n* = 3/group. *P vs NE, *P* < 0.05. **F**, **G**: Changes in β_1_-AR endocytosis caused by β_1_-AA in HL-1 cells with and without the overexpression of β_2_-AR-EYFP (β_2_-AR OE). Scale bar = 10 μm. *n* = 3/group. ^†^*P* vs β_1_-AA, *P* < 0.05. Data in **D**–**G** were analyzed by two-way ANOVA with the Bonferroni test. **H** The effects of β_1_-AA on intracellular Ca^2+^ in HL-1 cells with and without the overexpression of β_2_-AR-EYFP (β_2_-AR OE). Fluo-4 (10 μM) acted as a Ca^2+^ indicator. *n* = 3/group.

β_2_-AR regulates β_1_-AR signaling by interacting with it. To assess the possible role of β_2_-AR in β_1_-AA-restricted endocytosis, we first observed the effect of β_1_-AA on the binding of β_1_-AR to β_2_-AR. Purified human β_1_-AR and β_2_-AR proteins were isolated and used in the following experiment. The 300 nM purified β_2_-AR protein was coupled on the surface of a chip, and different concentrations (58.75 nM - 1880 nM) of purified β_1_-AR protein were added. Surface plasmon resonance (SPR) detection showed that purified β_1_-AR and β_2_-AR proteins could bind directly in a dose-dependent manner; the KD value was 2.147 μM (Fig. 3A, Table S17). β_1_-AA reduced the binding of β_1_-AR to β_2_-AR (Fig. 3B, Table S18) with an IC_50_ of 1.16 ± 0.7 μM (Fig. 3C, Table S19). Moreover, β_1_-AA did not bind directly to β_2_-AR. These results suggested that β_1_-AA might attenuate the regulatory effect of β_2_-AR on β_1_-AR.

To investigate the influence of the insufficient regulatory function of β_2_-AR on β_1_-AR endocytosis, endogenous β_2_-AR expression in HL-1 cells was inhibited by transfection with siRNA β_2_-AR (Fig. S5A, B, Fig. S15 D1, D2). As observed with TIRF, after stimulation with NE (10^−5^ M) for 5 min, 10 min, and 30 min, the fluorescence intensities of β_1_-AR-GFP on the cell membrane were reduced by approximately 1%, 5%, and 11%, respectively, in the siRNA β_2_-AR group, which was markedly lower than that of the siRNA control group (reduced by 6%, 27%, and 29%). This indicated that the lack of β_2_-AR restricted β_1_-AR endocytosis (Fig. 3D, E, Fig. S22 A, Table S20).

To further clarify the influence of β_2_-AR on β_1_-AA-induced limited β_1_-AR endocytosis, β_2_-AR-EYFP was overexpressed in HL-1 cells transiently transfected with β_1_-AR-GFP (Fig. S6A, B, Fig. S16 A1, A2). As detected by TIRF, β_1_-AA (10^−7^ M) administration decreased the fluorescence intensities of β_1_-AR-GFP by 14%, 24%, and 27% at 5 min, 10 min and 30 min after β_2_-AR overexpression, respectively. These results indicated the promoting effect of the increased β_2_-AR expression on β_1_-AA-induced β_1_-AR endocytosis (Fig. 3F, G, Fig. S22 B, Table S21). We further observed the impact of β_2_-AR on the sustained activation of β_1_-AR signaling. The β_2_-AR-EYFP plasmid was transfected into HL-1 cells, and a Fluo-4 fluorescence probe was used to identify intracellular Ca^2+^ changes. As shown in Fig. 3H (Table S22), compared with the nonoverexpression group, β_2_-AR-EYFP overexpression significantly reduced the amplitude increase in Ca^2+^ induced by β_1_-AA and delayed the time of increase in Ca^2+^. Transfection of β_2_-AR-EYFP alone or the addition of negative IgG had no effect on β_1_-AR endocytosis or Ca^2+^ levels in HL-1 cells. The above results suggested that a lack of β_2_-AR regulation might be responsible for weakened endocytosis and the long-term activation of β_1_-AR caused by β_1_-AA.

### Biased activation of β_2_-AR/Gi promotes β_1_-AR endocytosis and then inhibits the persistent activation of β_1_-AR elicited by β_1_-AA

**Fig. 4:**
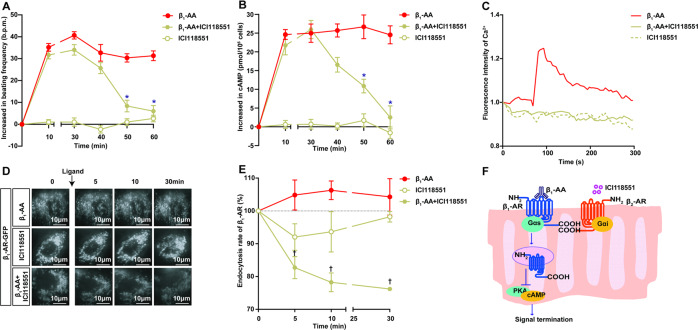
By activating β_2_-AR/Gi, ICI118551 promoted β_1_-AA-induced β_1_-AR endocytosis and prevented continuous β_1_-AR activation. **A**, **B** The impact of ICI118551 on β_1_-AA-induced increased beating frequency and cAMP level of NRCMs, respectively. ICI118551 was added 10 min after administration of β_1_-AA. *n* = 3–6/group. *P vs β_1_-AA + ICI118551, *P* < 0.05 (two-way ANOVA with Bonferroni test). **C** The reversal effect of ICI118551 on the persistent rise in intracellular Ca^2+^ caused by β_1_-AA in HL-1 cells. HL-1 cells were pretreated with Fluo-4 for 90 min, ICI118551 was added, and then β_1_-AA was added after 5 min. *n* = 3/group. **D** and **E** The reversal effect of ICI118551 on the limited endocytosis of β_1_-AR caused by β_1_-AA. Scale bar = 10 μm. *n* = 3–6/group. ^†^*P* vs β_1_-AA, *P* < 0.05 (two-way ANOVA with Bonferroni test). **F** illustrates that β_2_-AR/Gi-biased activation by ICI118551 could reverse the limited endocytosis and sustained activation of β_1_-AR induced by β_1_-AA.

**Fig. 5:**
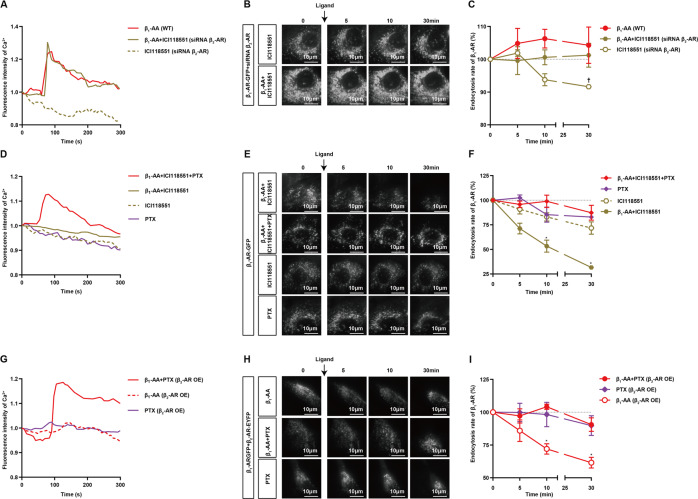
siRNAβ_2_-AR or PTX blocked the endocytosis-promoting effect of β_2_-AR/Gi-signaling pathway for β_1_-AR. **A** The effect of ICI118551 on β_1_-AA-induced Ca^2+^ change in HL-1 cells after interfering with β_2_-AR (siRNA β_2_-AR). *n* = 3/group. Because the experiments in Fig. 4C and Fig. 5A were done at the same time, the same β_1_-AA (WT) group in Fig. 4C were re-used as control here. **B** and **C** The effect of ICI118551 on β_1_-AA restricting β_1_-AR endocytosis in HL-1 cells which were transfected with siRNA β_2_-AR. Scale bar = 10 μm. *n* = 3–6/group. **C** the β_1_-AA (WT) group represented that β_1_-AA stimulated β_1_-AR overexpressed HL-1 cells. Because the experiments in Fig. 4E and Fig. 5C were done at the same time, the same β_1_-AA (WT) group in Fig. 4E were re-used as control here. **D** The effect of ICI118551 on β_1_-AA-induced Ca^2+^ change in HL-1 cells pretreated with PTX (1.5 μg/ml, 13 hours). *n* = 3/group. **E**, **F** The effect of ICI118551 on β_1_-AA restricting β_1_-AR endocytosis in HL-1 cells, which were pretreated with PTX. Scale bar = 10 μm. *n* = 3/group. **G** The effects of β_1_-AA on intracellular Ca^2+^ when the HL-1 cells overexpressed β_2_-AR-EYFP (β_2_-AR OE) and pretreated with PTX. *n* = 3/group. **H**, **I**, The change of β_1_-AR endocytosis caused by β_1_-AA after the HL-1 cells overexpressing β_2_-AR-EYFP (β_2_-AR OE) and pretreated with PTX. Scale bar = 10 μm. *n* = 3/group. *P vs β_1_-AA + PTX, *P* < 0.05. Data of **C**, **F**, and **I** were analyzed by two-way ANOVA with Bonferroni test. **B**, **E** and **H** were the representative images. **C**, **F** and **I** were the quantitative graphs, respectively.

Accumulating reports have demonstrated that β_2_-AR negatively regulates β_1_-AR by coupling with Gi. We demonstrated that as a specific β_2_-AR antagonist, ICI118551 (10^−5^ M) also acted as a biased agonist of the β_2_-AR/Gi-signaling pathway (Fig. S7A and B, Fig. S16 B1-B3). This effect could be inhibited by the Gi inhibitor pertussis toxin (PTX, 1.5 μg/ml, pretreatment for 13 h) (Figure S7C and D, Figure S17 A1 and A2). The cultured NRCMs were treated with β_1_-AA (10^−7^ M) with or without ICI118551 (10^−5^ M). As shown in Fig. 4, the β_1_-AA-induced increased beating frequency and the intracellular cAMP content of NRCMs were significantly relieved by ICI118551 supplementation, but ICI118551 alone had no effects on these parameters (Fig. 4A, B, Table S23 and S24). Furthermore, the continuous increase in intracellular Ca^2+^ induced by β_1_-AA disappeared after preincubation with ICI118551 in HL-1 cells (Fig. 4C, Table S25). Then, HL-1 cells transfected with β_1_-AR-GFP were used to detect β_1_-AR endocytosis via TIRF. After HL-1 cells were preincubated with ICI118551 for 5 min and subsequently stimulated with β_1_-AA, β_1_-AR-GFP fluorescence was reduced by 17%, 21%, and 24% at 5 min, 10 min, and 30 min, respectively, indicating marked β_1_-AR endocytosis (Fig. 4D, E, Fig. S22 C, Table S26). However, the above effects related to ICI118551 supplementation disappeared after interference with the expression of endogenous β_2_-AR (Fig. 5A–C, Table S27, Fig. S23A, Table S28) or with PTX (1.5 μg/ml) treatment (Fig. 5D–F, Table S29, Fig. S23B, Table S30) in HL-1 cells. We further treated HL-1 cells overexpressing β_2_-AR with PTX. The results showed that after PTX inhibited Gi, the effects of β_2_-AR overexpression on promoting β_1_-AR endocytosis and inhibiting the intracellular Ca^2+^ increase caused by β_1_-AA disappeared (Fig. 5G–I, Table S31, Fig. S23C, Table S32). To identify the role of β_2_-AR/Gi-signaling in NE-induced endocytosis of β_1_-AR, we tested the β_1_-AR endocytosis in HL-1 cells overexpressed Gi by TIRF. The results showed that overexpression of Gi did not enhance the endocytosis of β_1_-AR upon NE stimulation (Fig. S8A, B, Fig. S24 A, Table S33). In summary, activating the β_2_-AR/Gi-signaling pathway reversed β_1_-AA-induced weakened β_1_-AR endocytosis, leading to the termination of β_1_-AR signaling over time (Fig. 4F).

### β_2_-AR/Gi promotes β_1_-AR endocytosis by activating GRK2

**Fig. 6:**
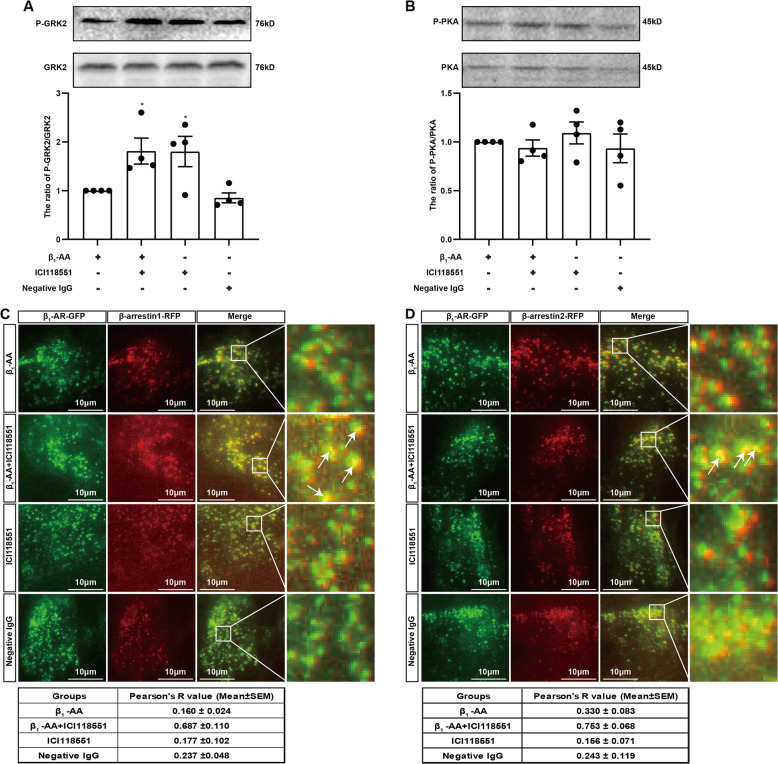
Activation of GRK2 enhanced the recruitment of β_1_-AR and β-arrestin stimulated by β_1_-AA. **A** The phosphorylation of GRK2 was detected after HL-1 cells were stimulated with ICI118551 alone or in combination with β_1_-AA for 5 min. *n* = 4/group. *P vs β_1_-AA, *P* < 0.05 (one-way ANOVA with Dunnett test). **B** The detection of PKA phosphorylation. *n* = 4/group. **C**, **D** TIRF was used to detect the recruitment of β_1_-AR and β-arrestin1/2 (above pictures), and Pearson’s coefficient (below tables) was calculated to reflect the colocalization between the two proteins. White arrows indicate the colocalization of β_1_-AR with β-arrestin1 (**C**) or β-arrestin2 (**D**). *n* = 3/group.

β-arrestin1/2 initiates GPCR endocytosis after binding to receptors phosphorylated by intracellular protein kinases. G protein-coupled receptor kinase 2 (GRK2) and protein kinase A (PKA) are two predominant kinases that promote β_1_-AR phosphorylation in cardiomyocytes. To explore the mechanism of promoting β_1_-AR endocytosis after the biased activation of β_2_-AR/Gi, we tested the activities of GRK2 and PKA in HL-1 cells. As shown in Fig. 6, both β_1_-AA (10^−7^ M) and ICI118551 (10^−5^ M) costimulation and ICI118551 single stimulation increased GRK2 phosphorylation (Fig. 6A, Fig. S17 B1, B2), whereas negative IgG (10^−7^ M) stimulation showed no significant effect (Fig. 6A). However, neither β_1_-AA or ICI118551 alone nor their combination affected PKA phosphorylation (Fig. 6B, Fig. S18 A1, A2). These results indicated that GRK2 activation might promote the recruitment of β-arrestin and β_1_-AR, subsequently triggering β_1_-AR endocytosis. Therefore, we detected the recruitment of β_1_-AR-GFP and β-arrestin1/2-RFP on the surface of the HL-1 cell membrane by TIRF. The results showed that owing to the activity of ICI118551, β_1_-AA stimulation increased the recruitment of β_1_-AR and β-arrestin. During β_1_-AA stimulation, the Pearson coefficients (Pearson’s *R* values) that measured the colocalization of β_1_-AR with β-arrestin1 and β-arrestin2 were 0.160 ± 0.024 and 0.330 ± 0.083 as the mean ± SEM, respectively, whereas in the ICI118551 plus β_1_-AA stimulation group, the Pearson coefficient was increased to 0.687 ± 0.110 (β-arrestin1) and 0.753 ± 0.068 (β-arrestin2) as the mean ± SEM (Fig. 6C, D). In addition, after interference with GRK2 expression in HL-1 cells, the reversal effect of ICI118551 on β_1_-AA-restricted β_1_-AR-GFP endocytosis disappeared (Fig. S9 A-D, Fig. S18 B1, B2, Fig. S24 B, Table S34). These results suggested that after the biased activation of the β_2_-AR/Gi-signaling pathway, ICI118551 could activate GRK2, thereby increasing the recruitment of β_1_-AR and β-arrestin, promoting receptor endocytosis and terminating the continuous activation of β_1_-AR under the action of β_1_-AA.

**Fig. 7:**
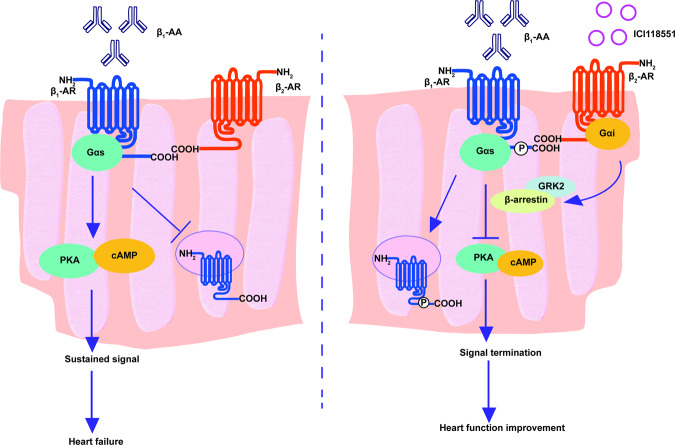
Work model. Under the action of β_1_-AA, the sustained activation of β_1_-AR is due to the decrease of endocytosis. Biased activation of β_2_-AR/Gi/GRK2 signaling pathway can promote the endocytosis of β_1_-AR that restricted by β_1_-AA, leading to the termination of β_1_-AA-induced continuous activation of β_1_-AR and improvement of cardiac structure and function.

### Biased β_2_-AR/Gi activation ameliorates cardiac structural damage and dysfunction in β_1_-AA-positive mice

To verify that activating the β_2_-AR/Gi-signaling pathway protected β_1_-AA-induced cardiac injury in vivo, wild-type Balb/c male mice (6–8 weeks old) without β_1_-AA were divided into four groups: saline (3.7 μg/kg, once a week, i.p.), β_1_-AA (10 μg/kg, every 2 weeks, i.p.), and therapy groups β_1_-AA combined with ICI118551 and ICI118551 alone (32.7 μg/kg, once a week, i.p.) (Fig. S10A). Eight weeks later, the β_1_-AA levels detected by enzyme-linked immunosorbent assay were significantly higher in the β_1_-AA and therapy groups than in the saline control group (Fig. S11, Table S35). M-mode echocardiography detection showed that the left ventricular ejection fraction (LVEF) decreased by 12.6% after 8 weeks of β_1_-AA treatment, and the left ventricular end-diastolic diameter (LVEDD) and left ventricular end systolic diameter (LVESD) increased by 15% and 42%, respectively. However, LVEF, LVESD, or LVEDD in the saline control group and the ICI118551 treatment group showed no significant change compared with preadministration measures (Fig. S10B, C, Table S36). Hematoxylin–eosin (HE) staining showed broken myocardial fibers in the β_1_-AA group, which were significantly alleviated after treatment with ICI118551 (Figure S10D). These results suggested that β_1_-AA caused cardiac dysfunction and structural damage in mice, and ICI118551, a biased agonist of β_2_-AR/Gi, had a certain therapeutic effect on cardiac injury caused by β_1_-AA.

## Discussion

Previous studies have shown that β_1_-AA promotes the occurrence and development of HF by continuously activating β_1_-AR. However, the specific mechanism is not fully understood. Receptor endocytosis is one of the important mechanisms for terminating signal transduction. The main study findings were that β_1_-AA continuously activated β_1_-AR by restricting β_1_-AR endocytosis and that biased activation of the β_2_-AR/Gi pathway could activate GRK2, promote β_1_-AR endocytosis restricted by β_1_-AA in vitro and alleviate the morphologic and functional damage in the mouse heart in vivo Fig. 7.

β_1_-AA is produced against β_1_-AR-ECII [16]. In 1987, Wallukat et al. [17] found this autoantibody in dilated cardiomyopathy for the first time. Subsequently, high positivity rates of β_1_-AA have been detected in a variety of cardiovascular diseases, especially in HF [3, 18, 19]. Therefore, the relationship between β_1_-AA and HF has gradually attracted researchers’ attention. β_1_-AA elicits various pathological effects, such as triggering cardiomyocyte apoptosis [20], reducing the effective refractory period of the atrium and inducing atrial fibrillation [18], and inducing calcium overload [21], to cause cardiac damage. It is worth noting that continuously activating β_1_-AR is a common mechanism underlying these effects. Elucidating the related mechanism is conducive to drug development and clinical treatment. As an agonistic ligand control, NE was selected. It is an endogenous α_1_/β_1_-AR selective agonist belonging to the catecholamine category.

As a GPCR, the activation of β_1_-AR is a complicated process. The agonist ligand binds to β_1_-AR and induces its structural change, which in turn exposes the Gs protein-binding sites. The Gs protein undergoes the GTP cycle to convert to GTP-Gs and binds to β_1_-AR. Then, GTP-Gs activates AC and mediates signal transduction through cAMP-PKA [9, 22, 23]. To avoid the cytotoxicity caused by sustained GPCR activation, activated β_1_-AR needs to be desensitized and endocytosed over time. GPCR desensitization is divided into transient desensitization and long-term desensitization. Transient desensitization proceeds through the interaction of β-arrestin and GPCRs over a short period of time (minutes). Long-term desensitization proceeds through GPCR degradation in the lysosome over a long period of time (hours or days), during which the mRNA level of GPCR is reduced [24]. In this study, all experiments were completed in a short time; thus, receptor desensitization involved transient desensitization. The process is roughly as follows: GRK phosphorylates activated β_1_-AR, and then, β-arrestin1/2 in the cell recognizes and binds to phosphorylated β_1_-AR, blocking the binding between Gs protein and β_1_-AR and promoting the endocytosis of β_1_-AR [24, 25]. β-arrestin is a key protein in receptor endocytosis. Upon receptor activation, β-arrestin1/2 promotes receptor desensitization and endocytosis by binding to phosphorylated receptors. Overexpression of β-arrestin has been proved to be an effective way to enhance their recruitment to the receptor and promote receptor endocytosis [26]. In the present study, overexpressing β-arrestin promoted β_1_-AA endocytosis restricted by β_1_-AA in HL-1 cells.

To detect the β_1_-AR activation and endocytosis, NRCMs and HL-1 cells were used. NRCMs are rhythmic and their contraction profile just meets the needs of beating frequency experiments [27]. However, the rhythmic contraction will greatly disturb the TIRF observation and intracellular Ca^2+^ determination. Thus, a mouse myocardial HL-1 cell line, with good adhesion, was chosen as well. It has been studied that HL-1 cells can be used as a tool cell for β_1_-AR and β_2_-AR signaling pathways [28–30]. Here we confirmed that the proteins of β_1_-AR, β_2_-AR, β-arrestin1, and β-arrestin2 were expressed in HL-1 cells (Fig. S12A, Fig. S18 C1–D4). Furthermore, when given NE stimulation, the NRCMs need to be preincubated with Phe to block α_1_-AR, whereas HL-1 cells do not, because NE has a higher affinity for α_1_-AR than β_1_-AR in cardiomyocytes, whereas HL-1 cells do not express α_1_-AR (Fig. S12 B, Fig. S18 E1, E2).

In HL-1 cells, the change in cytosol Ca^2+^ upon NE stimulation was transient. However, a long time of elevated cytosol Ca^2+^ was observed under the β_1_-AA condition. The sustained high concentration of Ca^2+^ in the cytosol of cardiomyocyte was a fascinating phenomenon, which we cannot fully explain yet. We speculated that it may be owing to the change of activity of Ca^2+^ channels or calcium ATPase in sarcoplasmic reticulum when stimulated by β_1_-AA.

β_2_-AR is also important in cardiomyocytes because it can bind to either Gs or Gi protein [9, 31]. By coupling to the Gi protein, β_2_-AR inhibits AC activation to block β_1_-AR signaling and exerts cardioprotective effects [31]. In addition, β_2_-AR can target the C-terminus of β_1_-AR in a phosphodiesterase 4-dependent manner to limit signal diffusion to avoid cytotoxicity after β_1_-AR activation [32]. However, some studies found that the formation of the β_1_-β_2_-AR heterodimer strengthens the Gs signaling pathway in normal adult mouse cardiomyocytes and restricts β_2_-AR endocytosis [33, 34]. These reports point out the complexity of the relationship between β_1_-AR and β_2_-AR in different situations. Here, we verified that β_2_-AR could promote β_1_-AR endocytosis upon stimulation with either β_1_-AA or NE.

ICI118551 is used as a specific antagonist of β_2_-AR [35]. However, our study and other studies have found that ICI118551 preferentially activates β_2_-AR/Gi [10, 36]. ICI118551 is reported to have a protective effect on the cardiovascular system after activating the β_2_-AR/Gi pathway by reducing the contraction of myocytes in failing hearts [10] and reducing pulmonary artery tension [36]. We demonstrated that by activating the β_2_-AR/Gi pathway, ICI118551 could increase GRK2 activity, enhance the recruitment of β-arrestin1/2 to β_1_-AR, and improve β_1_-AA-induced β_1_-AR endocytosis. GRK2 is the predominant GRK and the primary regulator of β_1_-AR and β_2_-AR desensitization in the heart. GRK2 can phosphorylate activated β-adrenergic receptors, which are recognized by β-arrestin and initiate receptor endocytosis [37]. Contrary to expectation, we found that β_1_-AA upregulated β_1_-AR phosphorylation, whereas ICI118551 supplementation reduced it (Fig. S13A, B, Fig. S19 A1, A2). As reported, differences in receptor conformation stabilized by various ligands may expose different phosphorylation sites, which may elicit distinctive β-arrestin activation states and receptor endocytosis [38, 39]. We speculate that although β_1_-AR phosphorylation was decreased in the ICI118551 treatment group, the exposed phosphorylation sites were prone to coupling with β-arrestin. Of course, more work is needed to test this hypothesis.

Although the close relationship between β_1_-AA and HF is known, how to effectively block β_1_-AA remains difficult. β_1_-AR blockers are among the most commonly used medications in treating HF and the most convenient method because of their oral administration. However, β_1_-AR blockers cannot sufficiently block the continuous activation of β_1_-AR induced by β_1_-AA [40]. This problem was evident in this study, which may be owing to the differences in the binding sites of β_1_-AA and β_1_-AR blockers. Metoprolol recognizes the β_1_-AR ligand-binding pocket [23, 41, 42], whereas β_1_-AA binds to the second extracellular loop of β_1_-AR [16]. Animal studies have demonstrated that the combination of the β_2_-AR agonist fenoterol and β_1_-AR blocker metoprolol can improve the heart function of rats with HF. In addition, preferentially activating β_2_-AR/Gi can antagonize the apoptosis-promoting effect of β_1_-AR on cardiomyocytes through the PI3K-Akt pathway [43]. Here, we mainly found that activating β_2_-AR/Gi could exert a cardioprotective effect by promoting β_1_-AR endocytosis and terminating its signal in a timely manner. Therefore, biased activation of the β_2_-AR/Gi-signaling pathway may be a new strategy for the treatment of HF patients with β_1_-AA. However, the effectiveness of this program in clinical treatment needs to be further confirmed.

## Supplementary information

Supplemental material

Intracellular Ca2+ fluorescence intensity of HL-1 cells increased temporarily under NE stimulation.Intracellular Ca2+ fluorescence intensity of HL-1 cells increased temporarily under NE stimulation.

The fluorescence intensity of Ca2+ in HL-1 cells continuously increased after being treated by β1-AA.

Table S3

Table S4

Table S5

Table S6

Table S7

Table S8

Table S9

Table S10

Table S11

Table S12

Table S13

Table S14

Table S15

Table S16

Table S17

Table S18

Table S19

Table S20

Table S21

Table S22

Table S23

Table S24

Table S25

Table S26

Table S27

Table S28

Table S29

Table S30

Table S31

Table S32

Table S33

Table S34

Table S35

Table S36

## Data Availability

The original data sets are available from the corresponding author upon request.
