# Supplementary material for: Biased activation of β2-AR/Gi/GRK2 signal pathway attenuated β1-AR sustained activation induced by β1-adrenergic receptor autoantibody
Source: Cell Death Discov. 2021 Nov 8;7:340. doi: 10.1038/s41420-021-00735-2 (PMC8576015; doi:10.1038/s41420-021-00735-2)
Supplement: Supplementary file 1 — Supplemental material [file 41420_2021_735_MOESM1_ESM.docx]

**Supplementary Materials**

**Biased activation of β_2_-AR/Gi/GRK2 signal pathway attenuated β_1_-AR sustained activation induced by β_1_-adrenergic receptor autoantibody**

Hao Chen^a, #^, Ning Cao^a, b, #^, Li Wang^c^, Ye Wu^a^, Haojie Wei^a^, Yuming Li^d^, Youyi Zhang ^b^, Suli Zhang^a, e, *^, Huirong Liu^a, e, *^

^a^Department of Physiology & Pathophysiology, School of Basic Medical Sciences, Capital Medical University, Beijing 100069, PR China;

^b^Institute of Vascular Medicine, Cardiology Department, Peking University Third Hospital, Beijing 100191, PR China;

^c^Department of Pathology, School of Basic Medical Sciences, Shanxi Medical University, Taiyuan 030001, PR China;

^d^Department of Physiology & Pathophysiology, Yanjing Medical College, Capital Medical University, Beijing 101300, PR China;

^e^Beijing Key Laboratory of Metabolic Disorders Related Cardiovascular Disease, Capital Medical University, Beijing 100069, PR China.

**^#^These authors contributed equally to this work.**

***Address for correspondence:**

Huirong Liu, MD, PhD

Department of Physiology & Pathophysiology, School of Basic Medical Sciences, Capital Medical University

10 Xitoutiao, You An Men Street, Beijing City, 100069, China

Phone: +86-10-83911830

E-mail: [liuhr2000@ccmu.edu.cn](mailto:liuhr2000@ccmu.edu.cn).

Suli Zhang, PhD

Department of Physiology & Pathophysiology, School of Basic Medical Sciences, Capital Medical University

10 Xitoutiao, You An Men Street, Beijing City, 100069, China

Phone: +86-10-83950504

E-mail: [sueney716@126.com](mailto:sueney716@126.com)

This PDF file includes:

Supplementary materials and methods

Supplementary figures S1 to S24

Supplementary tables S1 to S36

Supplementary videos S1 to S2

Supplementary figures and videos legends

**Supplementary Materials and Methods**

**Patients and samples**

The behavior of collecting patients' peripheral blood complied with medical standards. There were 25 patients screened in this study, of which 12 were β_1_-AA-positive patients and 13 were negative patients. The informed consent was obtained from all patients. The selection criteria were: male; 50-80 years old; chronic heart failure; New York Heart Association (NYHA) class III-IV. The exclusion criteria were: autoimmune diseases; metabolic-related cardiomyopathy; malignant tumors; acute myocardial infarction; infectious diseases (the clinic data was attached to supplement file, Table S1). The β_1_-AA-positvie IgG and -negative IgG were purified and stored at -80 °C until assay.

**Cell culture**

HL-1 cells and HEK293 cells were preserved in our laboratory. HL-1 cells, NRCMs and HEK293 cells were cultured in incubator (37 °C, 5% CO_2_). The cell culture medium was formulated as 10% fetal bovine serum (Gibco, USA), Penicillin-Streptomycin (100 IU/ml, 15140122, Gibco, USA) in 50ml DMEM (Corning, USA).

**Cyclic adenosine monophosphate measurements**

Standard preparation: Seven tubes were labeled 2, 4, 8, 16, 32, 64, and 128 fmol, respectively. 500 μl of test buffer was added each other. 500 μl original acylated standard solution (2.56 pmol/ml) was added in the "128 fmol" tube and mixed uniform. 500 μl solution in the "128fmol" tube was taken into a "64 fmol" tube and mixed uniform. The remaining tubes were repeated the above dilution. 500 μl solution was discarded from the "2 fmol" tube. The volume of each standard solution was 500 μl. Sample detection:100 μl solution from the seven standards was added to the new tube as “Standard” tube. Other tubes were tagged "Control 1", "Control 2" and "Samples". Acetic anhydride and triethylamine were mixed evenly in 1: 2 as acylated reagents. The "Control 2" tube was added 500 μl test buffer. 500 μl sample solution that from cell lysate was taken into the "Samples" tube. 25 μl acylated reagents were added to “Standard” and "Samples” tubes. After adding the acylation reagent, each tube should be mixed immediately. Taking 100 μl solution from “Standard” and "Samples” tubes to new tubes separately. 100 μl antiserum was added to "Control 2", “Standard” and "Samples” tubes. 100 μl labeling reagent was added to each tube. After mixing, reaction at 4°C for 4 hours. 500 μl separation buffer was added to "Control 2", “Standard” and "Samples” tubes. After mixing, reaction at room temperature for 10min. Centrifugation at 2000g for 10 minutes. The supernatant was discarded. The radioactivity count of each tube was measured.

**Cell transfection and Western blot analysis**

HL-1 cells were transiently transfected with β_1_-AR-GFP plasmid, or co-transfected with β-arrestin1/2-RFP or β_2_-AR plasmids, respectively, via Lipo2000 (Beijing likely biotechnology, China). After 24-hour, cells were subjected to corresponding experiments (protein expression, β_1_-AR endocytosis and intracellular Ca^2+^ detections). Small interfering RNA (siRNA) β_2_-AR (GCTGCAGAAGATAGACAAA) or GRK2 (CTGGACAAGAAACGCATCA) (50 nM, RIBOBIO, China) were transfected into HL-1 cell via Lipo2000. The cells were subjected to analysis (protein expression, β_1_-AR endocytosis and intracellular Ca^2+^ detections) randomly after 72-hour. In order to detect the recruitment of β_1_-AR and β-arrestin, HEK293 cells were transiently co-transfected with β_1_-AR-EYFP plasmid (Beijing likely biotechnology, China) and β-arrestin1-Rluc/β-arrestin2-Rluc plasmid via Lipo2000 (Invitrogen, 11668019, USA), respectively. After 24 hours of transfection, cells were subjected to analysis the protein expression by fluorescence and recruitment of β-arrestin to β_1_-AR by BRET. Western blot was performed per standard method, and proteins were visualized by Millipore Immobilon Western Chemiluminescent HRP Substrate (Billerica, MA, USA). Band intensity was determined by Image Lab software version 3.0 (Bio-Rad Laboratories, Hercules, CA, USA).

**Surface plasmon resonance**

Binding interactions between β_1_-AR and β_2_-AR were measured using Biacore T200 (GE, USA). In brief, 300 nM human recombinant β_2_-AR pure protein (Abacm, ab157862, Britain) was immobilized on a CM-5 sensor chip via an amine coupling reaction to a response of 2000 resonance units (RU) according to the manufacturer’s instructions. In the steady state direct binding experiments, β_2_-AR pure protein was immobilized onto the chip surface to achieve an RU of 525. The analyte, human recombinant β_1_-AR + Gαs fusion pure protein (Abcam, ab90827, Britain), was injected over the chip at various concentrations (58.75-1880 nM). The observed dissociation binding constant, KD, was calculated by using the average RU under steady state conditions. Data were fitted globally by using the steady state model provided by Biocore T200 software.

In competition assays, human recombinant β_2_-AR pure protein was immobilized onto a CM-5 sensor chip to a response of 525 RU as described above. A final concentration of 1880 nM human recombinant β_1_-AR pure protein was mixed with each β_1_-AA at various concentrations (117.5-1880 nM) in PBS-EP and the mixture was injected. The binding response and competitive inhibition reaction between β_1_-AR and β_2_-AR pure proteins were assessed using the equilibrium binding portion of the curves. Biocore T200 software was utilized to analyze the data, determine the affinity constant and the 50% competitive inhibition constant (IC_50_).

**Immunoprecipitation**

Immunoprecipitation experiment was performed to determine the level of GTP-Gαi after ICI1118551 (Selleck, S8114, USA) and PTX (MCE, HY-112779, USA) stimulation and the level of β_1_-AR-GFP phosphorylation in HL-1 cells. Cells were harvested using cell lysis buffer (P0013J, Beyotime, China). Active Gαi antibody or GFP antibody were added and incubated overnight at 4 °C. The next day, protein A/G agarose beads (P2012, Beyotime, China) were added to the lysates and incubated for 4 hours at 4 °C. After collection and thrice washings with immunoprecipitation lysis buffer, agarose beads were boiled with 2× loading buffer for 10 mins. Proteins were separated by sodium dodecyl sulfate polyacrylamide gel electrophoresis (SDS-PAGE) and analyzed by Western blot using Gαi antibody and Phospho-(Ser/Thr) Phe antibody.

**Bioluminescence resonance energy transfer technology**

β_1_-AR-EYFP plasmid was transiently co-transfected into HEK293 cells with β-arrestin1-Rluc/β-arrestin2-Rluc plasmid, respectively. After 24 hours, the cells were transferred into 96-well plates (3603, Axygen, USA) and cultured for another 24 hours. Then the cells were subjected to norepinephrine (NE, TargetMol, USA ), β_1_-AA, and negative IgG stimulation prior to detection. The BRET effect was detected after the addition of coelenterazine (5 μM, S2011, Promega, USA). The BRET signal was evaluated by the ratio of emission light 530 nm (EYFP) to 480 nm (Rluc).

**β_1_-AA level detection**

β_1_-AA levels in mice and heart failure patients’ sera were determined by enzyme linked immunosorbent assay using a synthetic peptide corresponding to the sequence of the second extracellular loop of mouse β_1_-adrenoceptor (β_1_-AR-ECII, 197-222, HWWRAESDEARRCYNDPKCCDFVTNR). The similarity of the β_1_-AR-ECII amino acid sequence between human and mouse was 100%. The β_1_-AR-ECII peptides were synthetized by Jill biochemical (Shanghai, China) and dissolved in 0.1 mol/L Na_2_CO_3_ to make a 10 μM stock solution. Dilution ratios of human and mouse sera were 1:100 and 1:10, respectively. Goat anti-human (1:500, ZSGB-BIO, ZDR-5301, China) or anti-mouse IgG/HRP (1:500, ZSGB-BIO, ZDR-5307, China) served as a secondary antibody to detect β_1_-AA.

**Selection and preparation of IgG samples**

The β_1_-AA-positive or -negative total IgGs were isolated from the sera of samples via Protein G Agarose Columns (GE, 17-1128-01, USA) per manufacturer instructions. The concentration of total IgGs was determined via pierce BCA protein assay kit (Thermo, 23225, USA) and diluted to 1μM with phosphate buffer prior to use.

**Mice echocardiography**

Cardiac functions were detected in M-mode ultrasound in awaken mice. Prior to ultrasound, chest and abdominal hairs were removed by depilation agents. LVEF, LVESD and LVEDD were measured by Vevo2100 (FUJIFILM VisualSonics,Inc., Canada).

**Histological analysis**

Mice were sacrificed by 1% isoflurane through inhalation anesthesia. Each heart was removed and cut into 3 transverse slices. The middle section was fixed in 10% buffered formalin and embedded into paraffin. 4 μm-thick heat sections were stained with Hematoxylin-eosin (Coolaber, SL7070, China). Images were obtained via Pannoramic scan. Images were processed via CaseViewer.

**Figure S1**


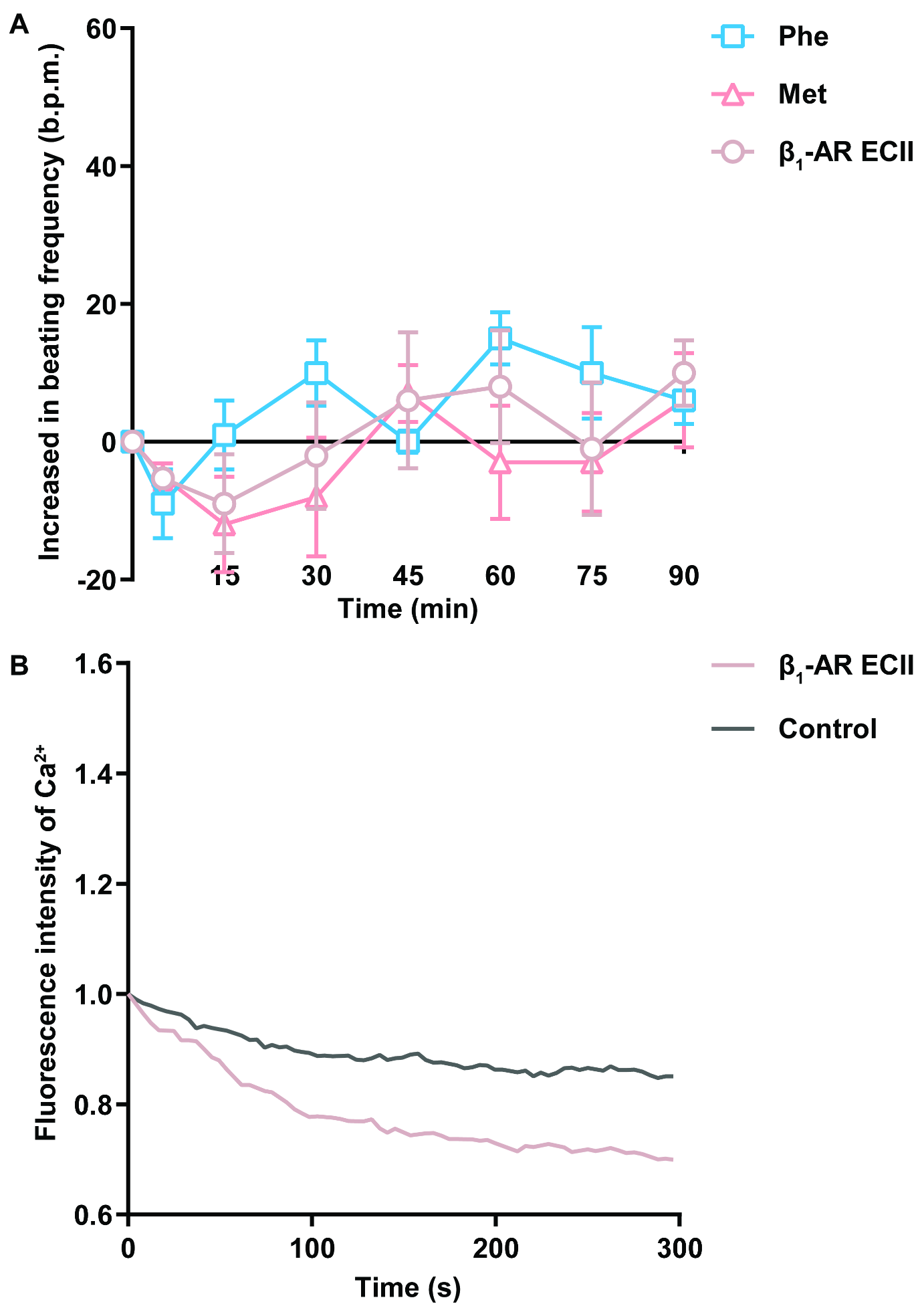


**Figure S2**


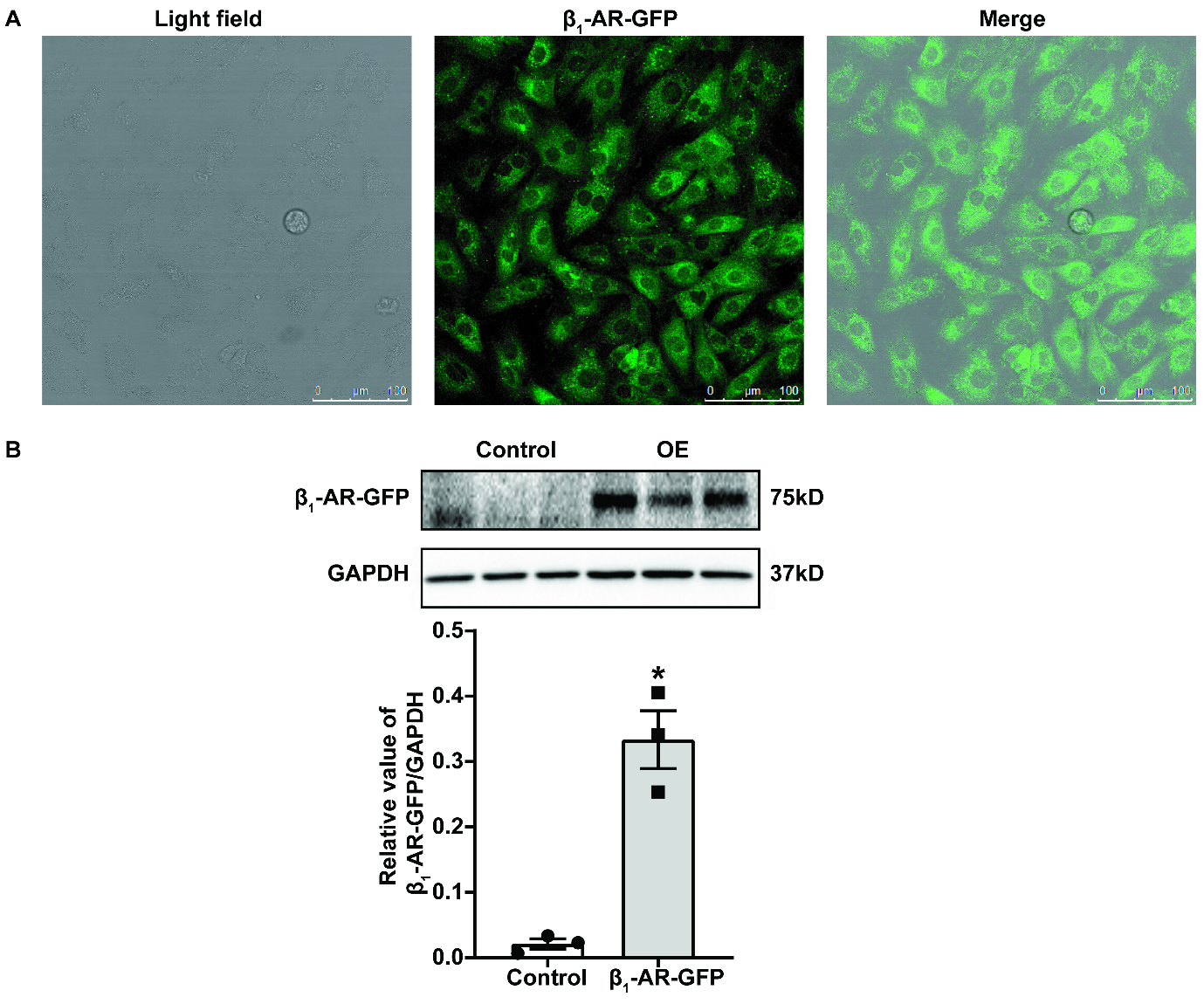


**Figure S3**


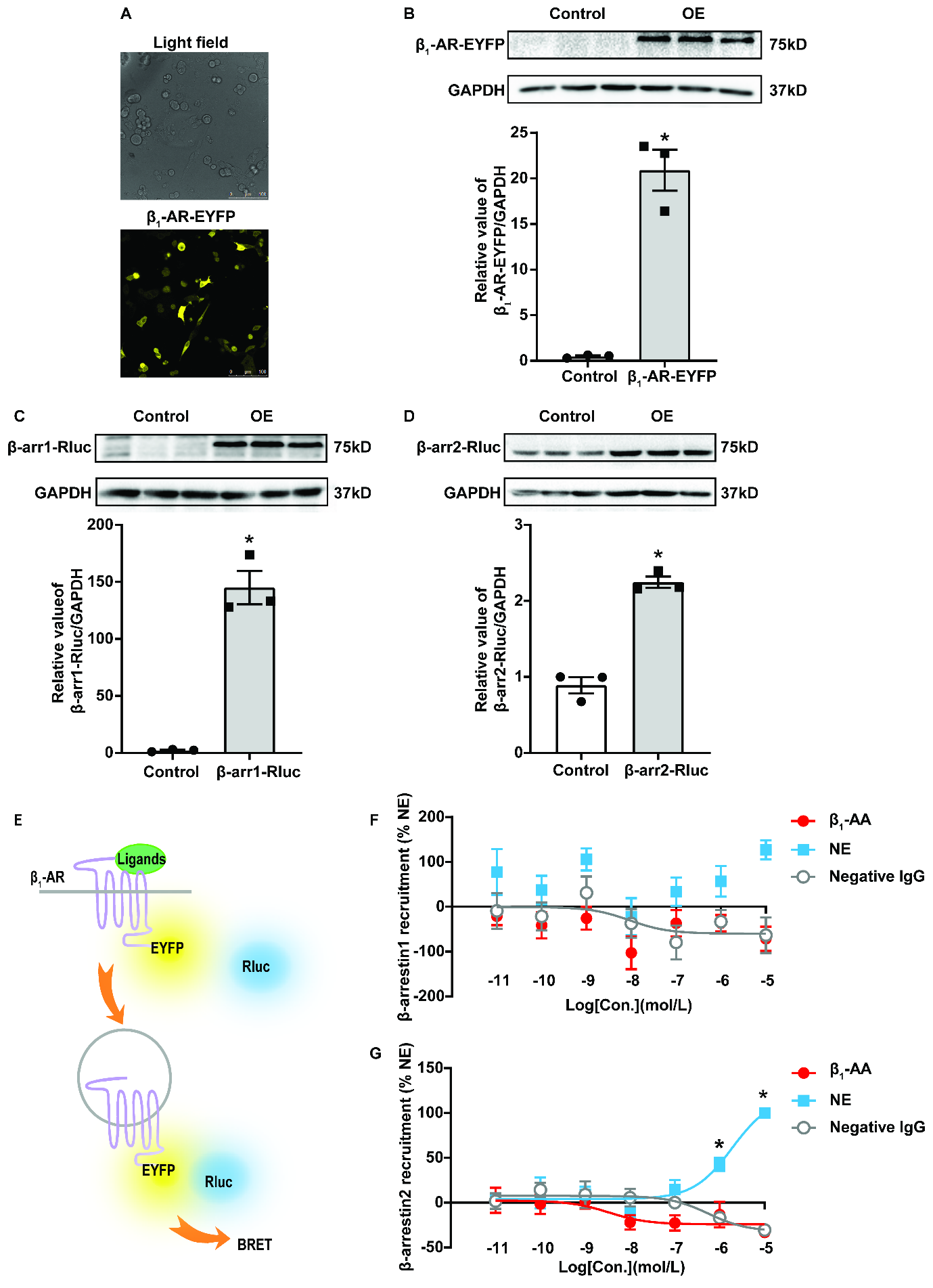


**Figure S4**


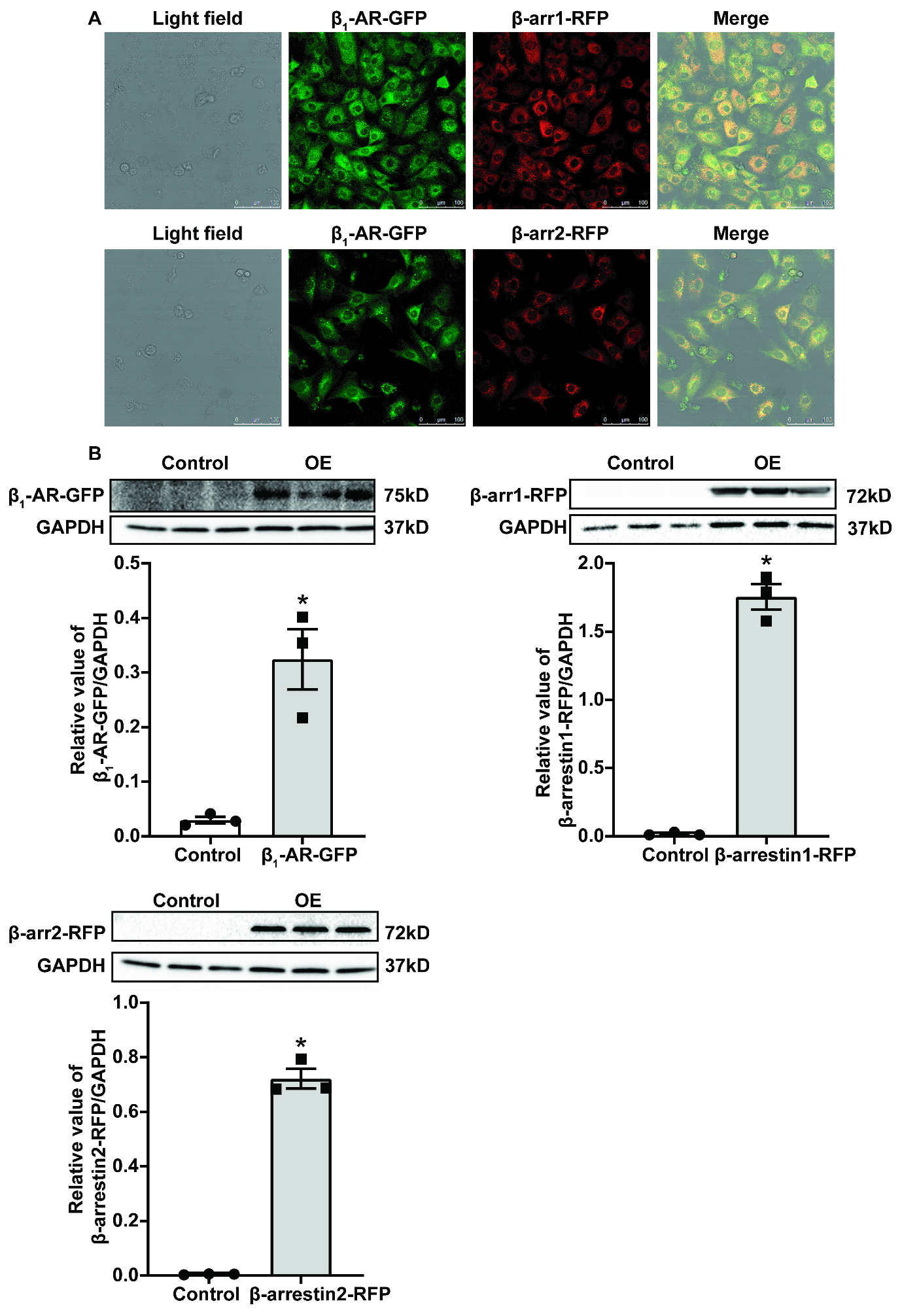


**Figure S5**


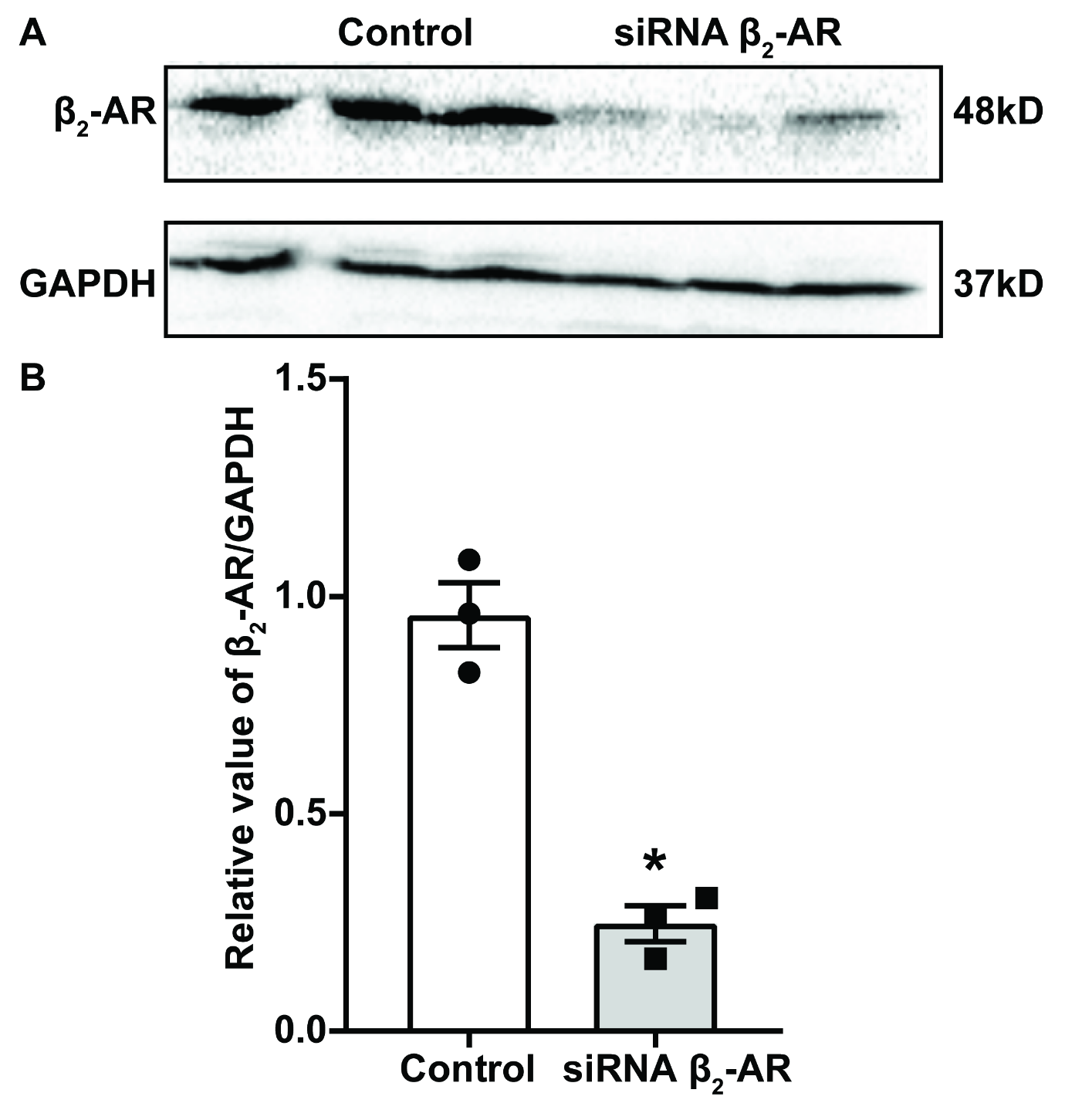


**Figure S6**


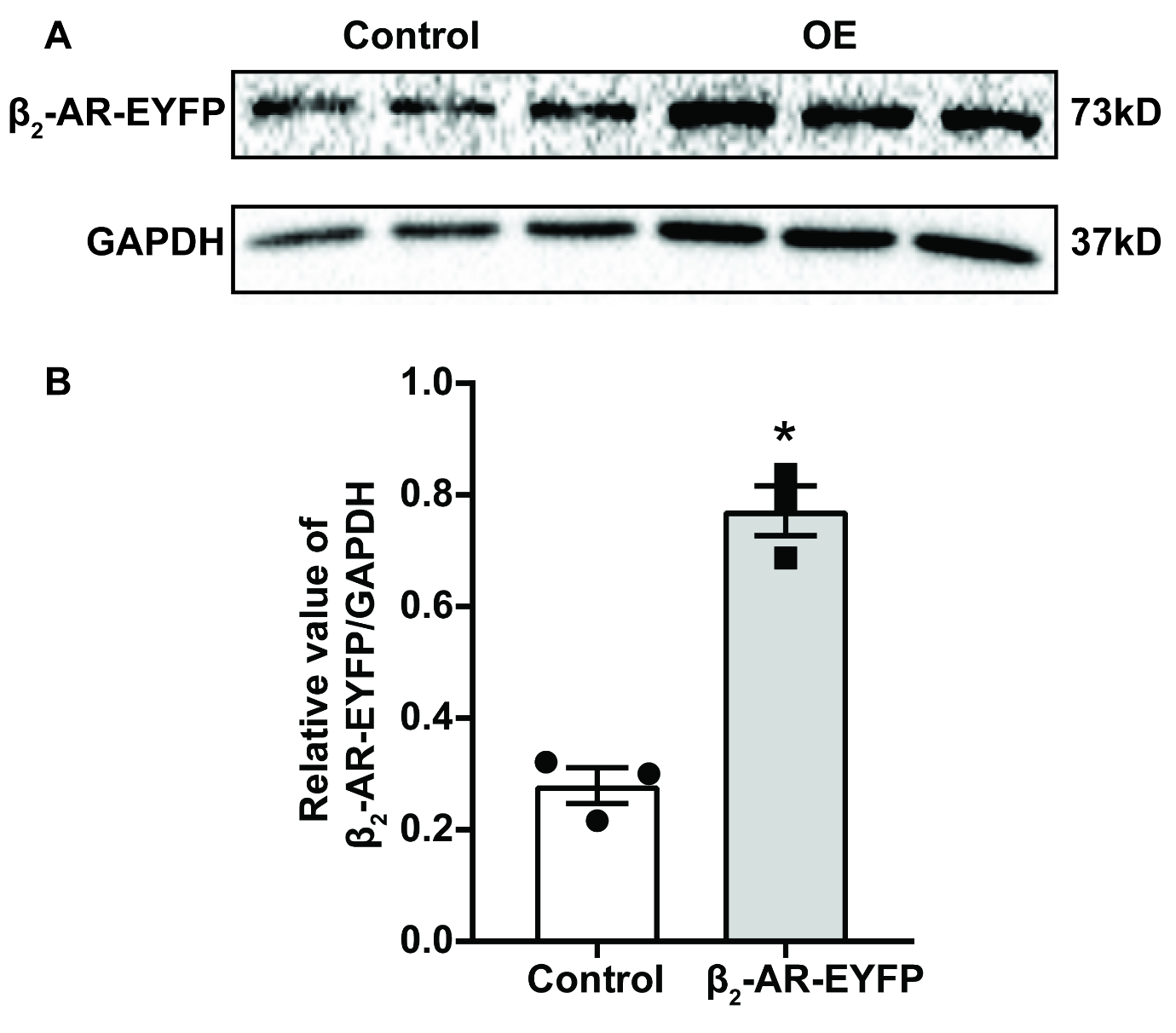


**Figure S7**


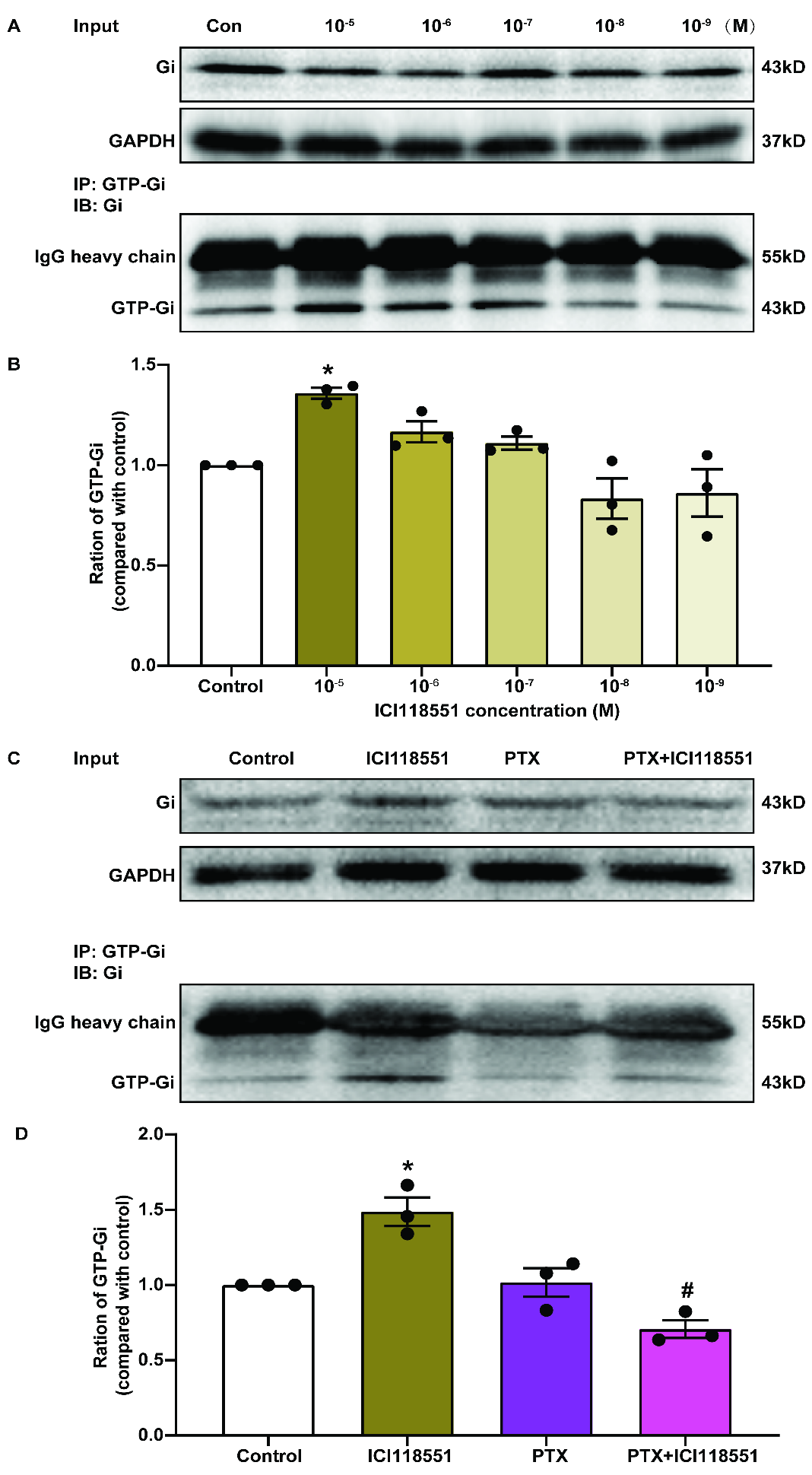


**Figure S8**

**
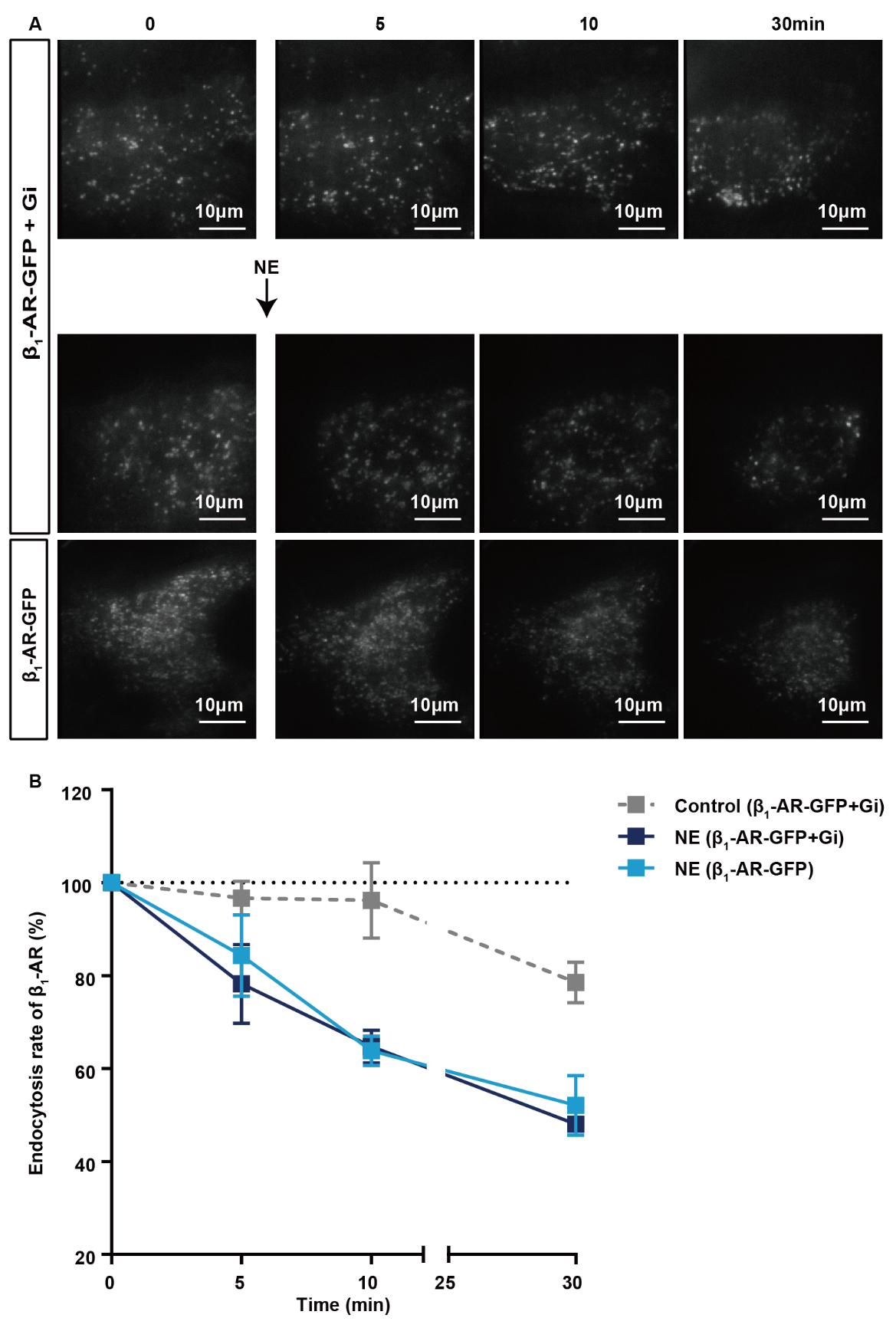
**

**Figure S9**


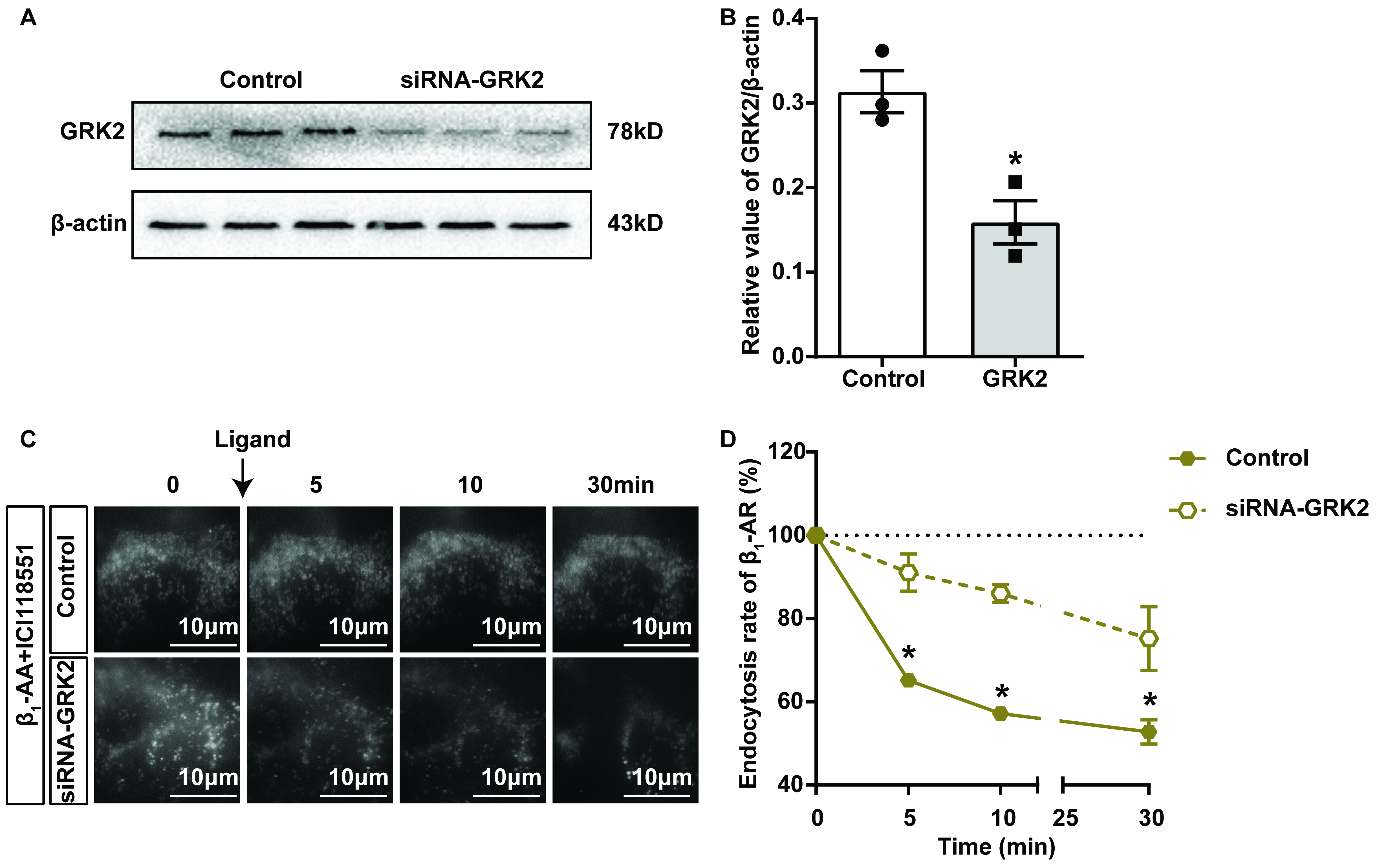


**Figure S10**

**
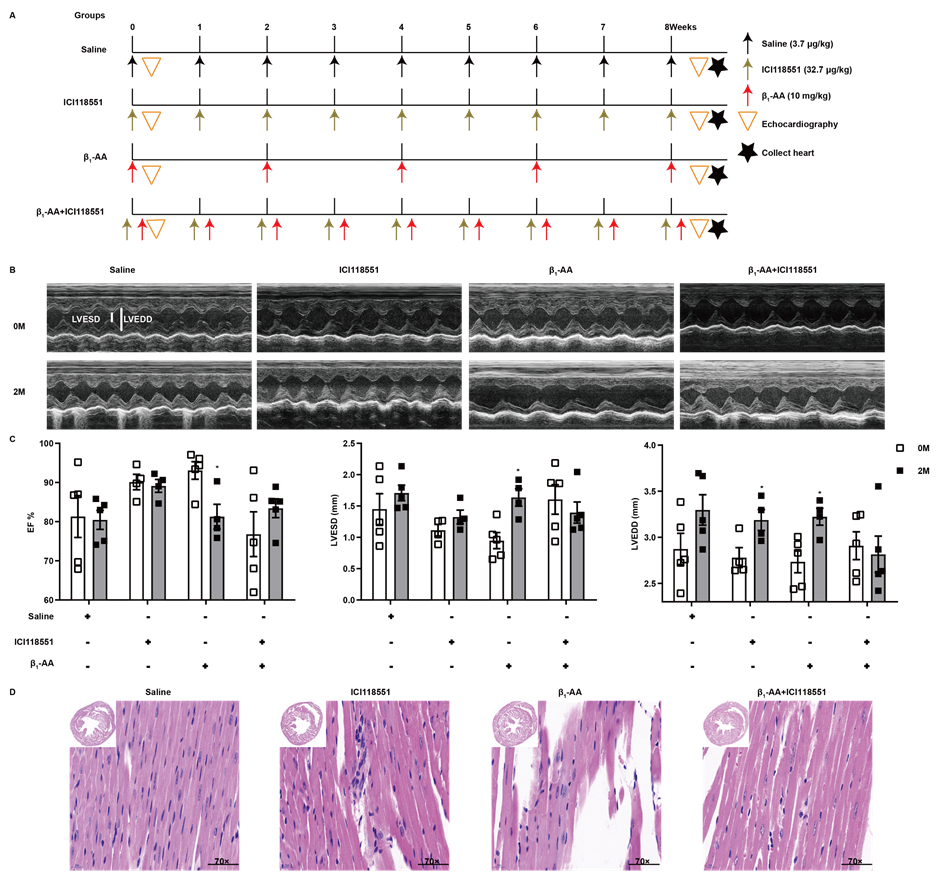
**

**Figure S11**


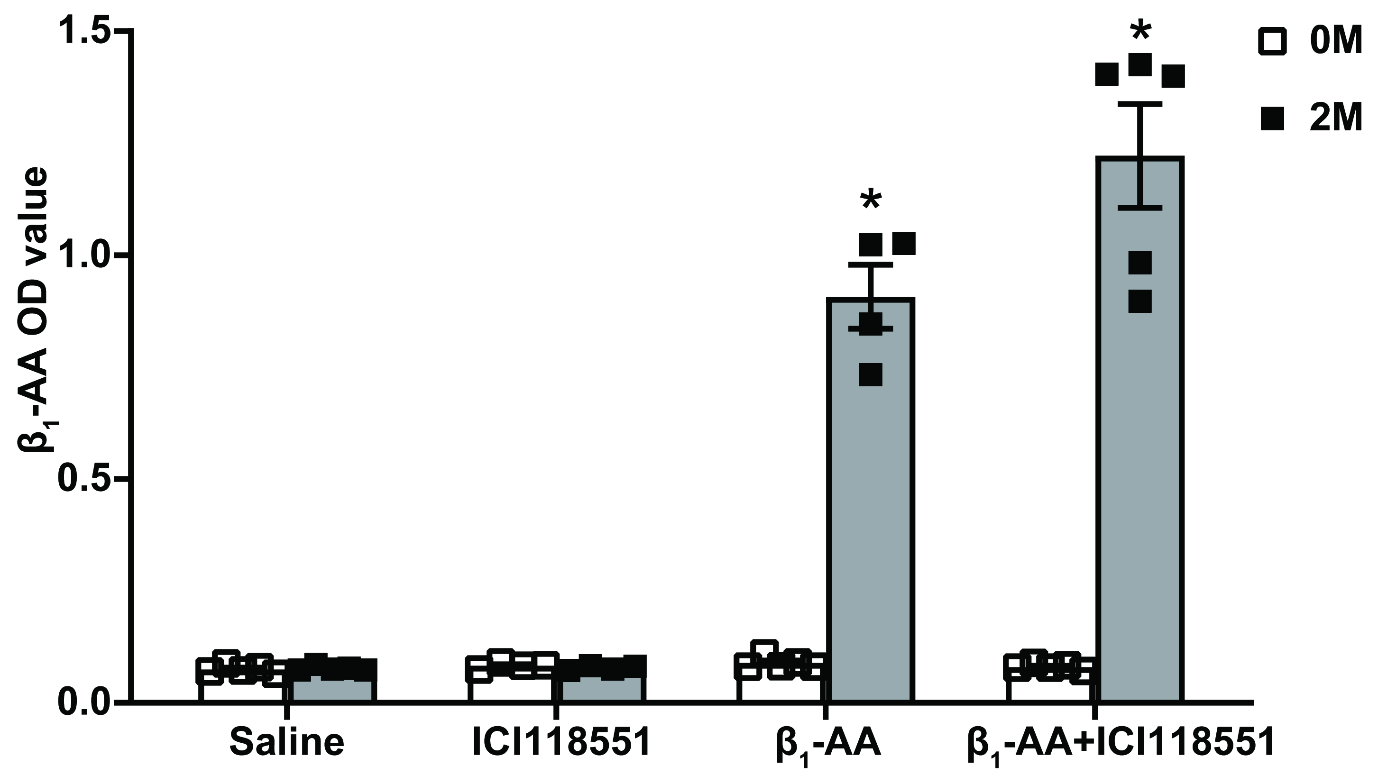


**Figure S12**

**
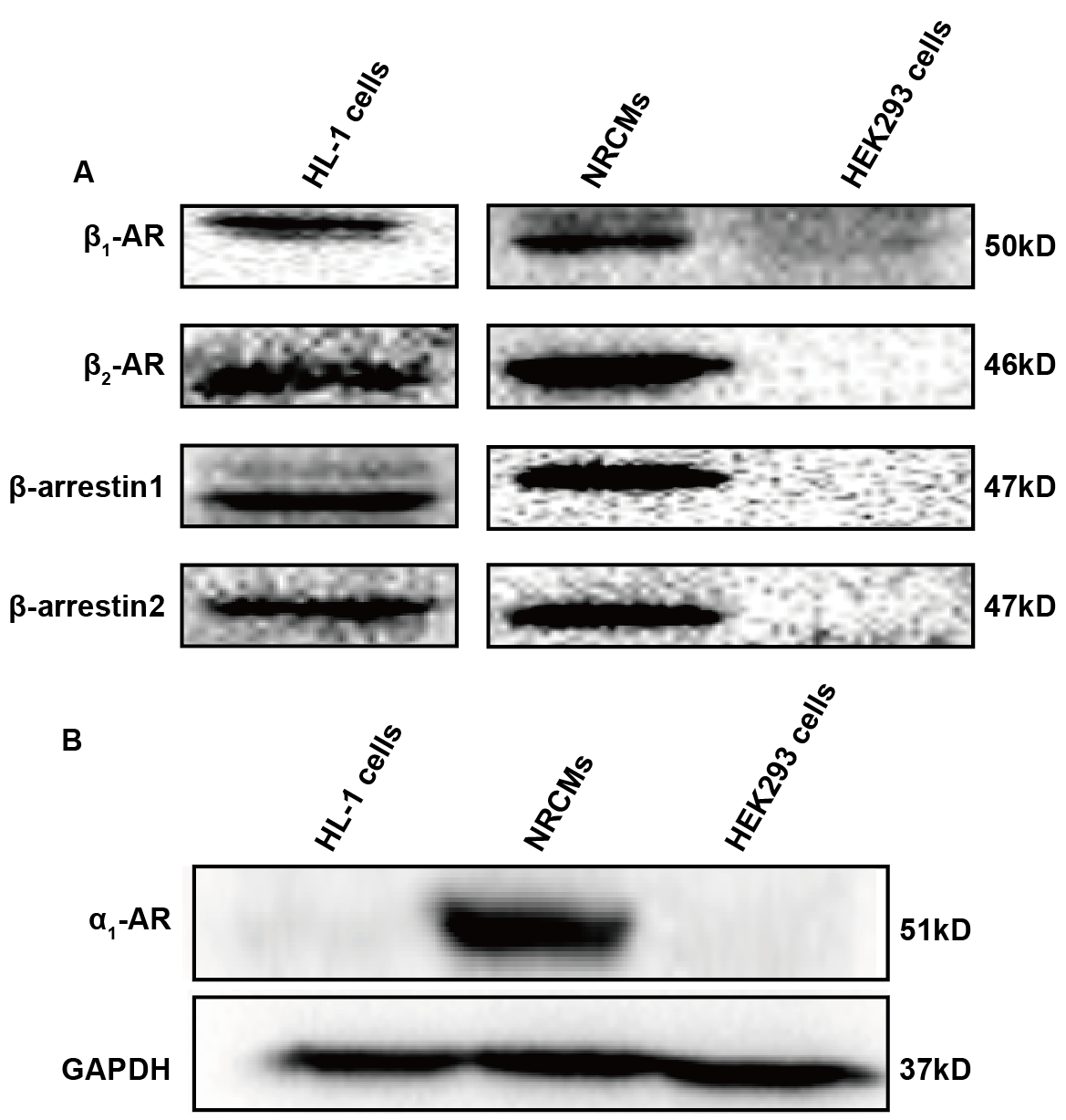
**

**Figure S13**


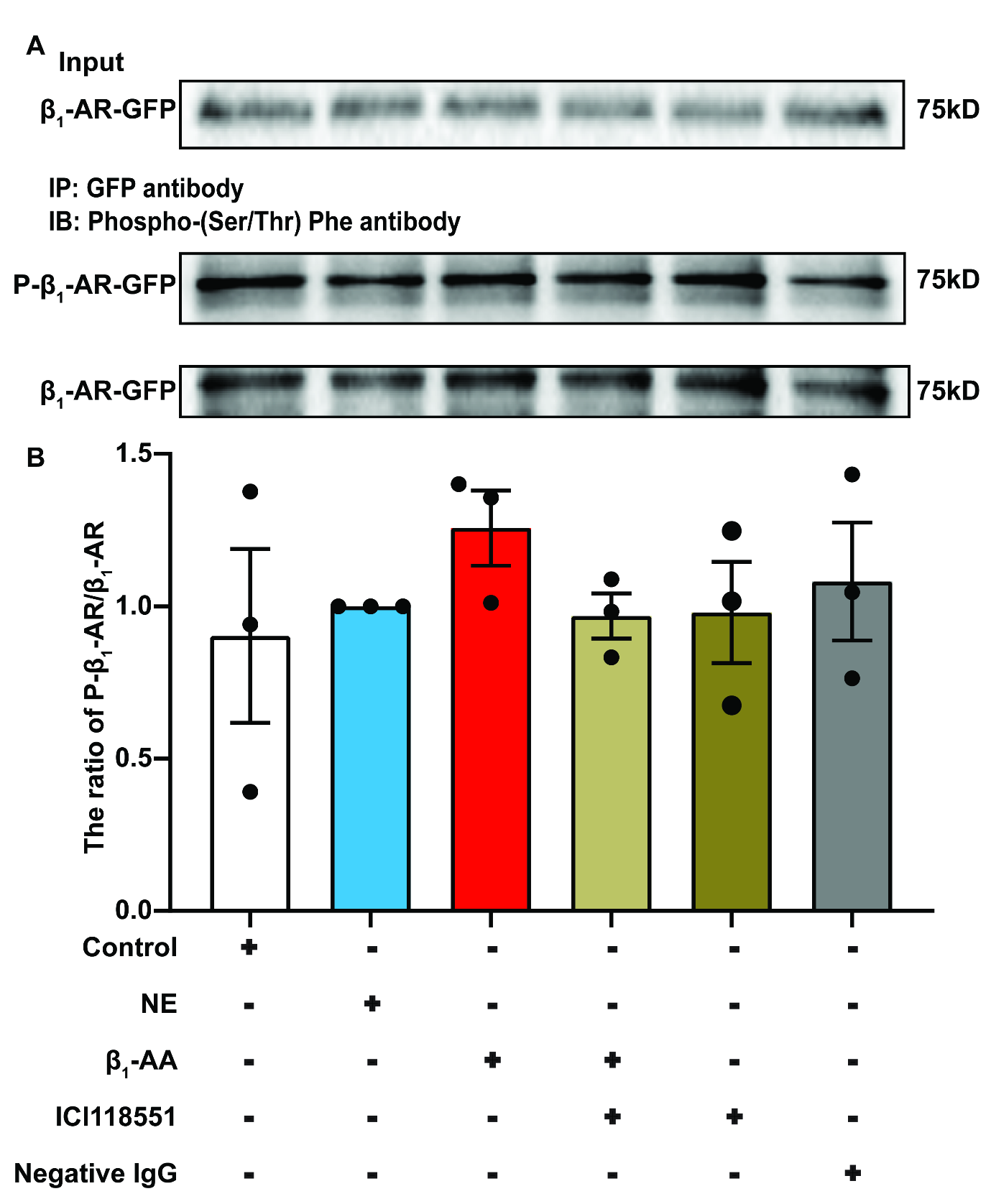


**Figure S14**


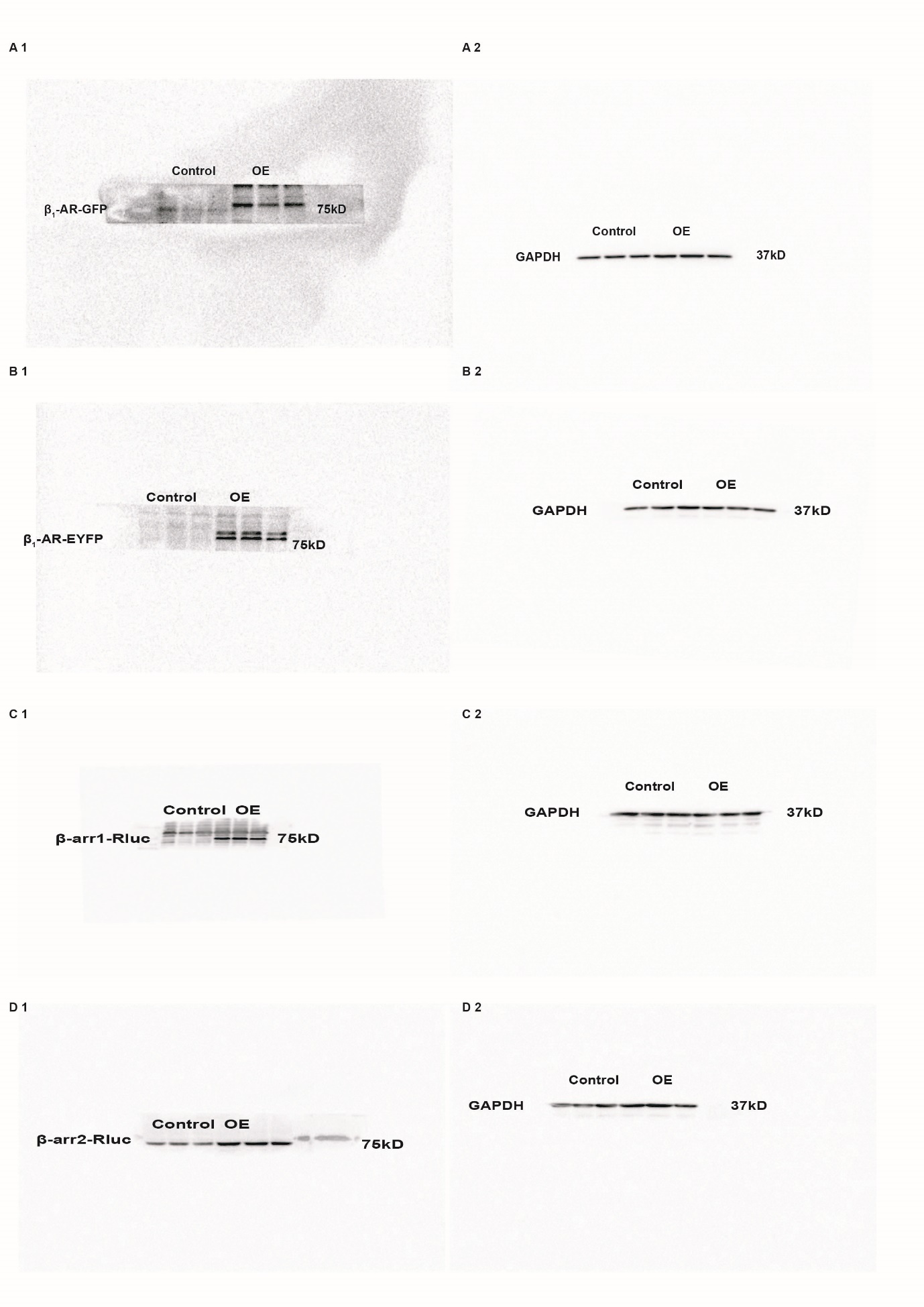


**Figure S15**


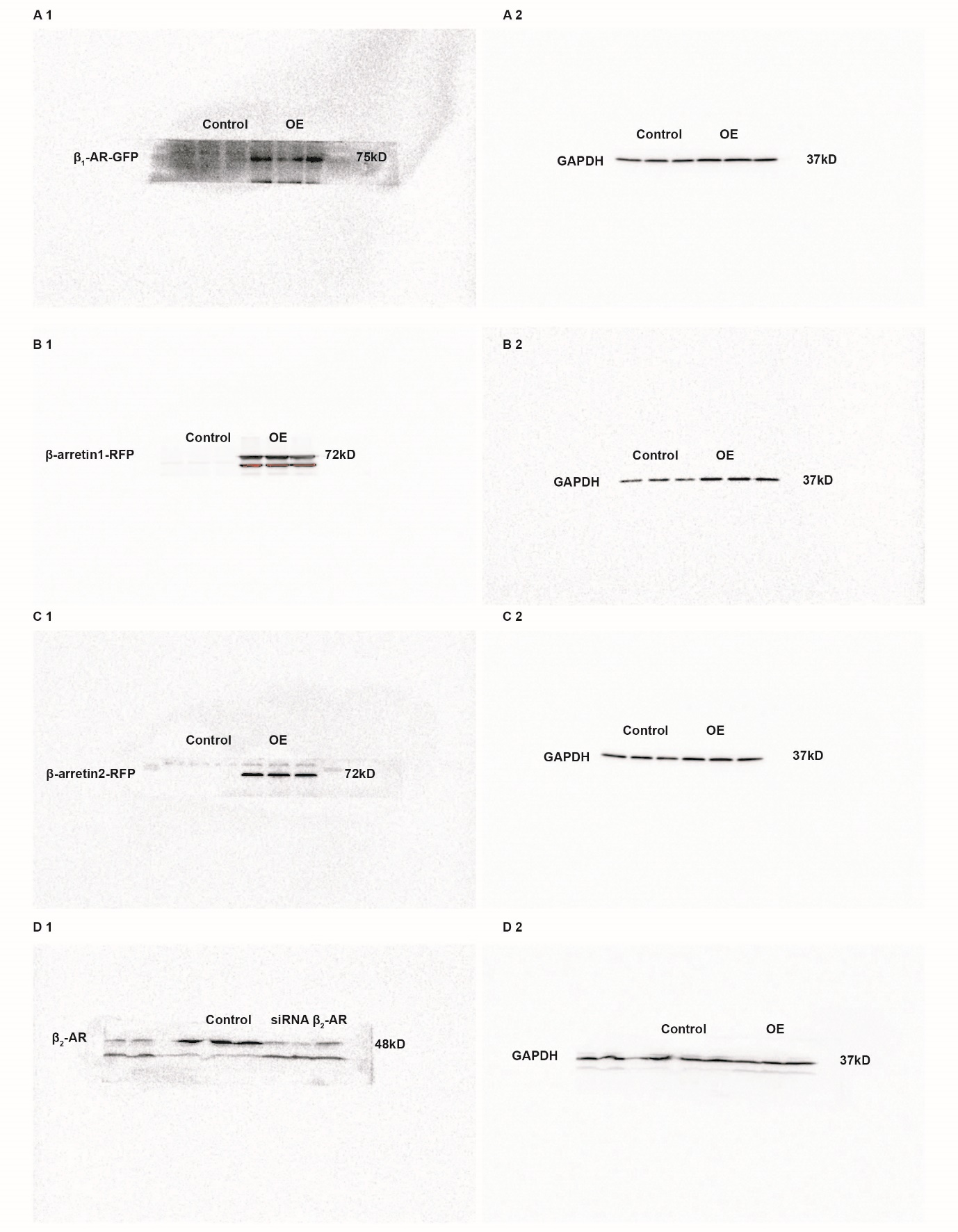


**Figure S16**


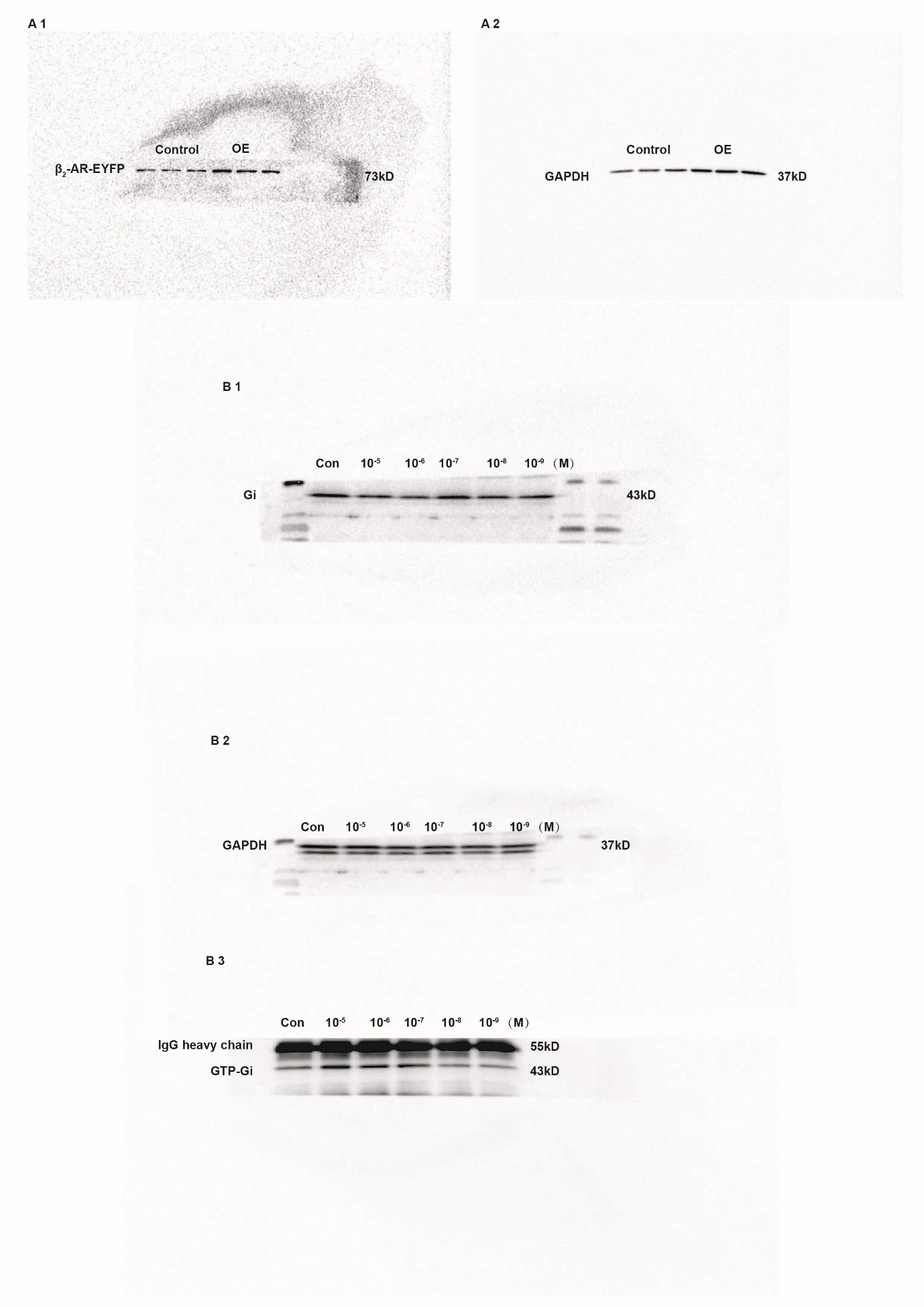


**Figure S17**

**
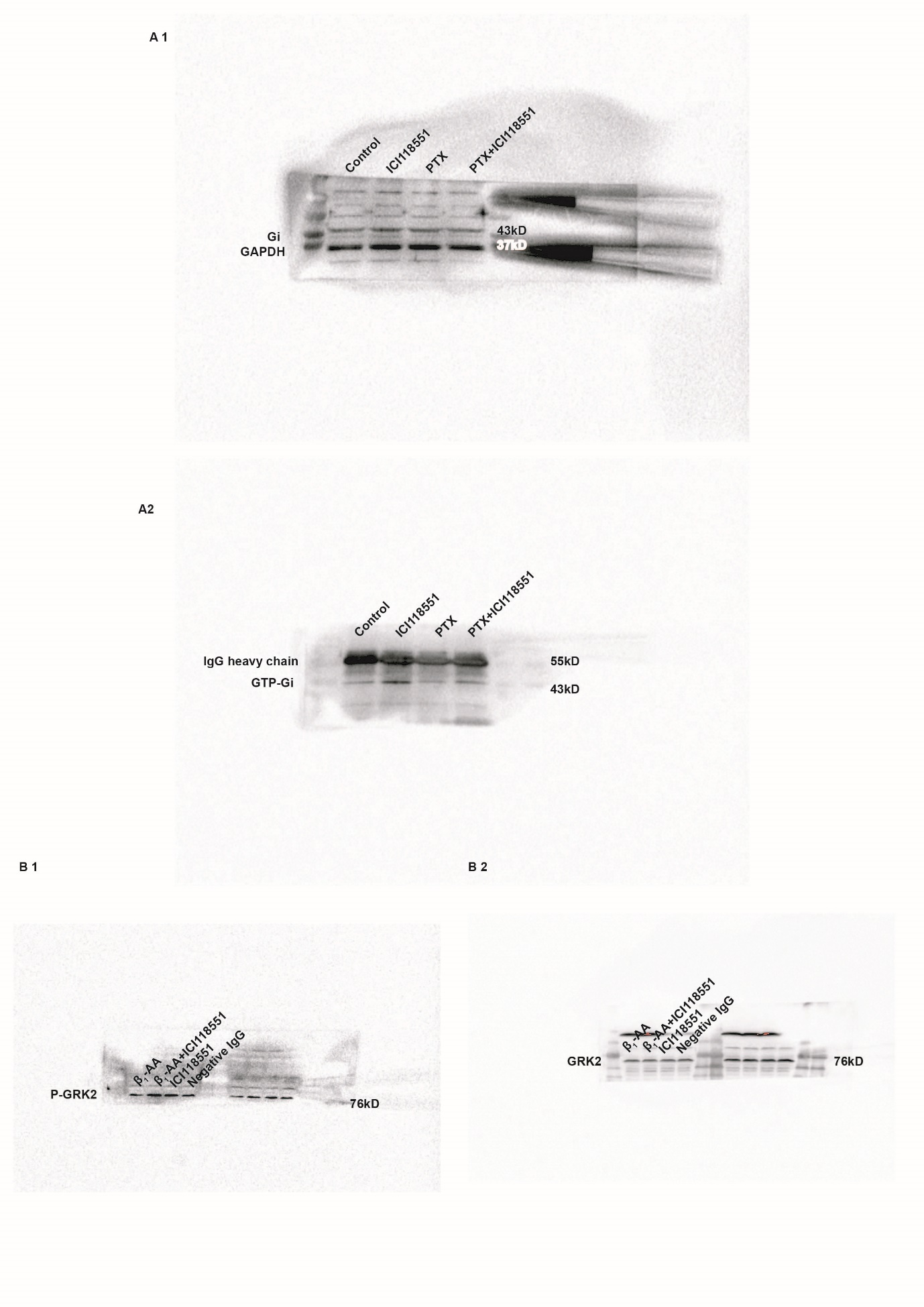
**

**Figure S18
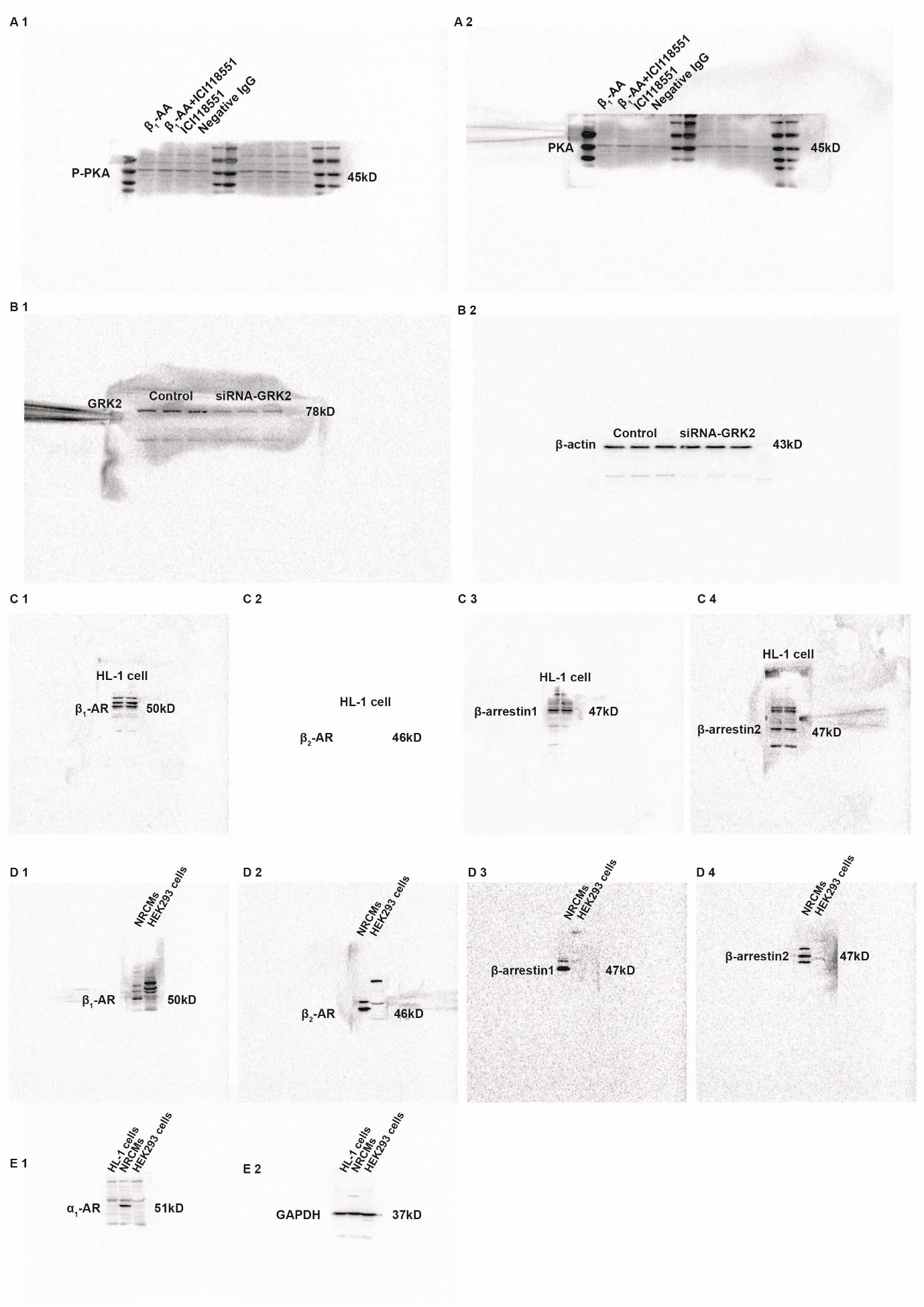
**

**Figure S19
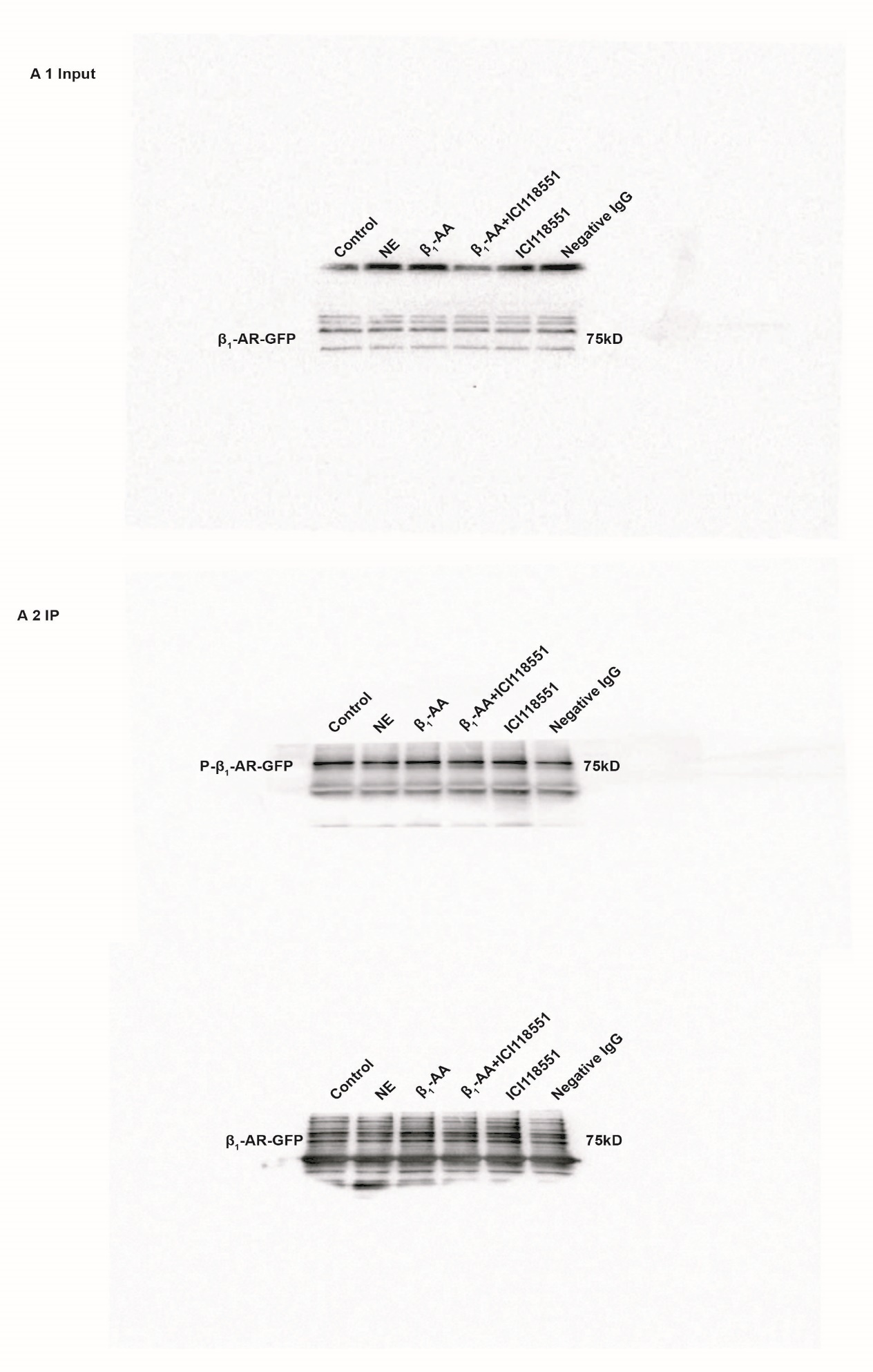
**

**Figure S20
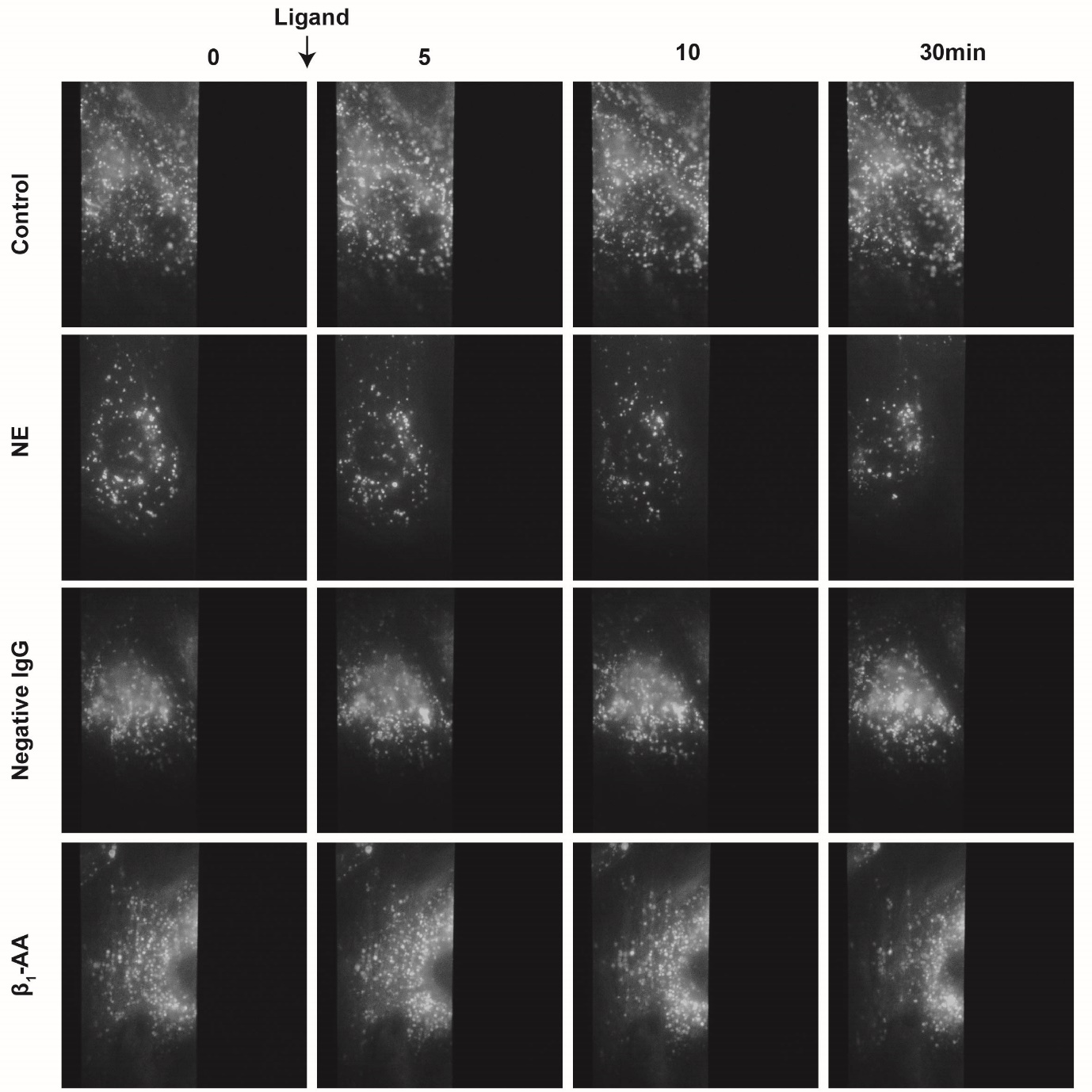
**

**Figure S21
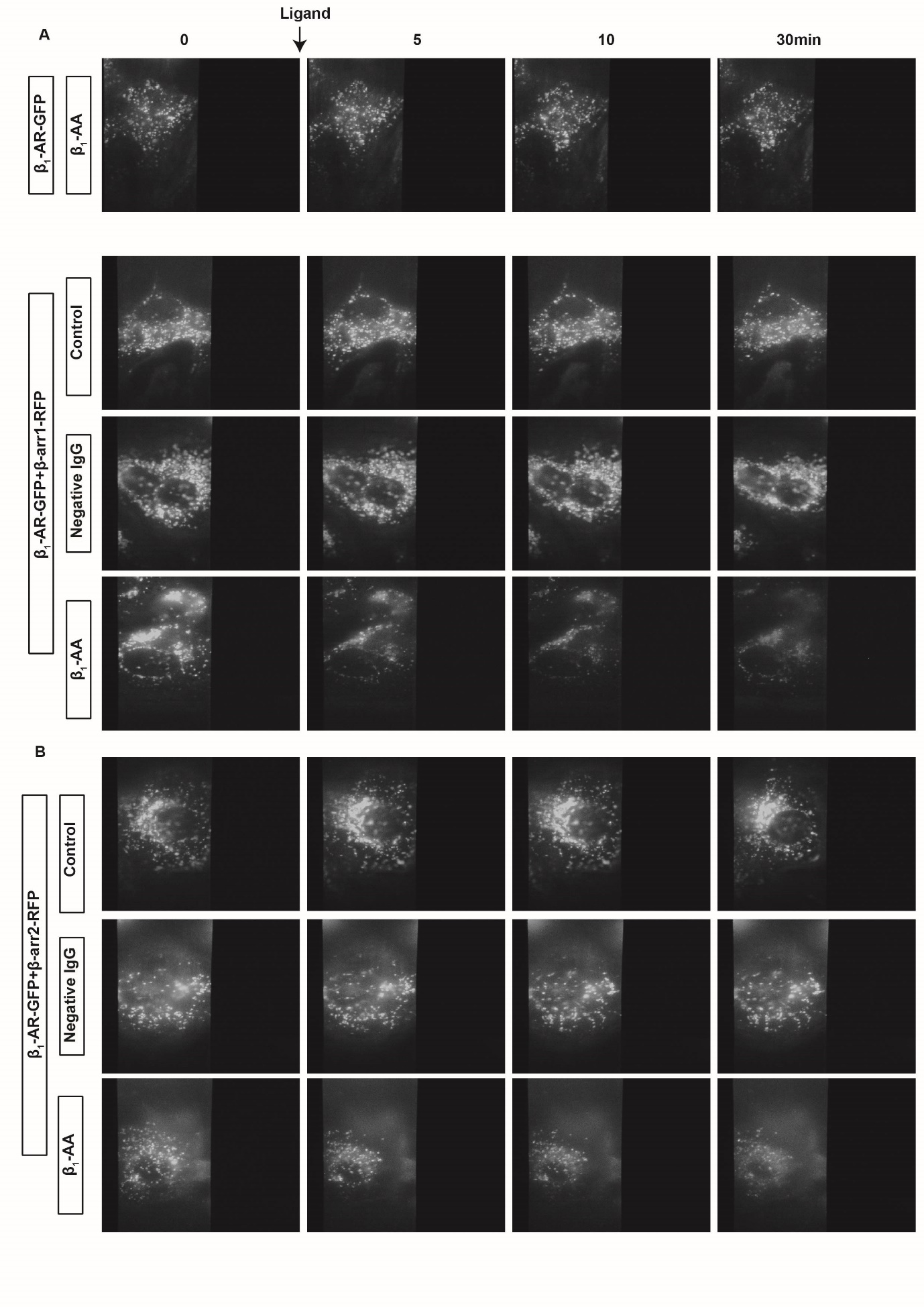
**

**Figure S22
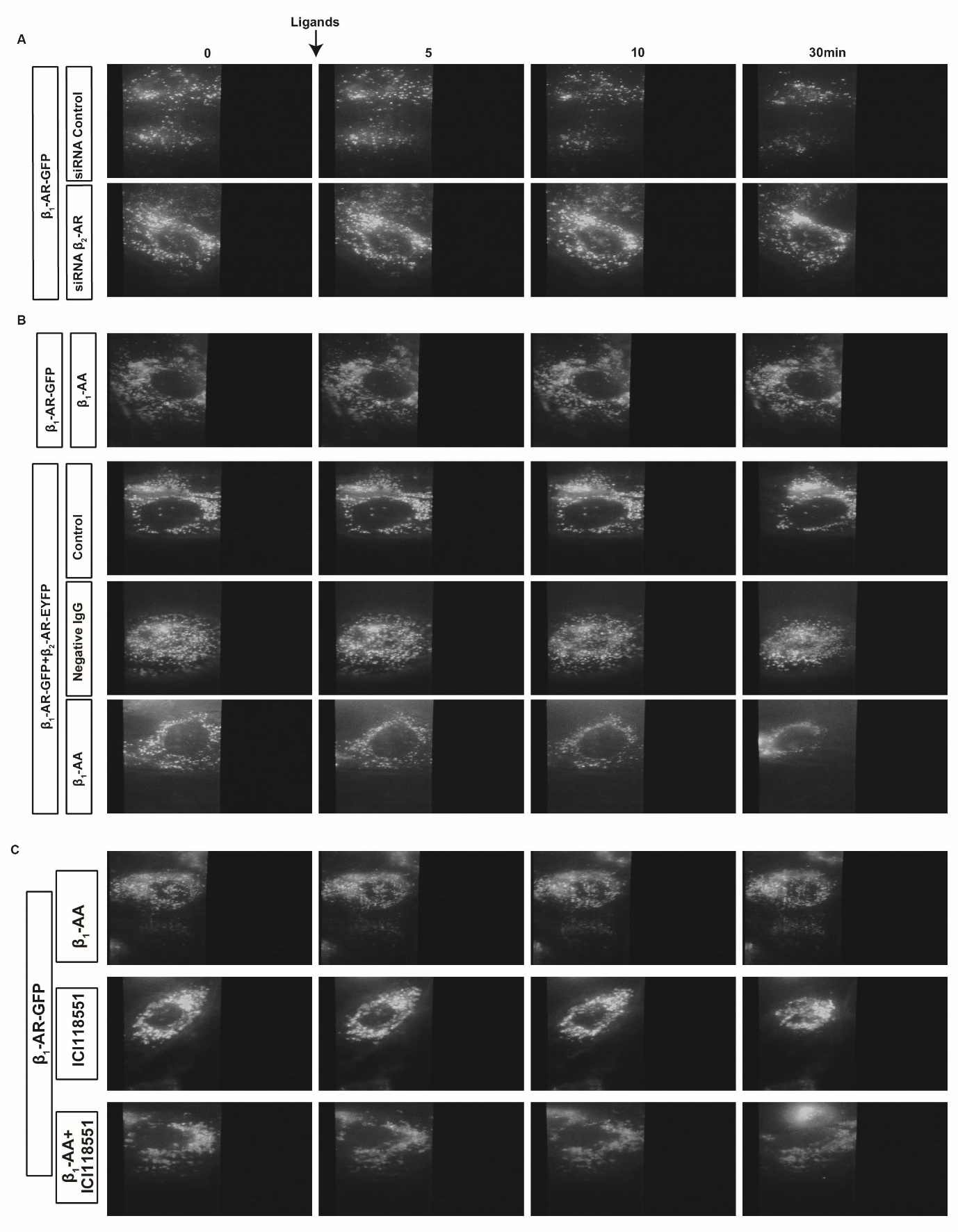
**

**Figure S23
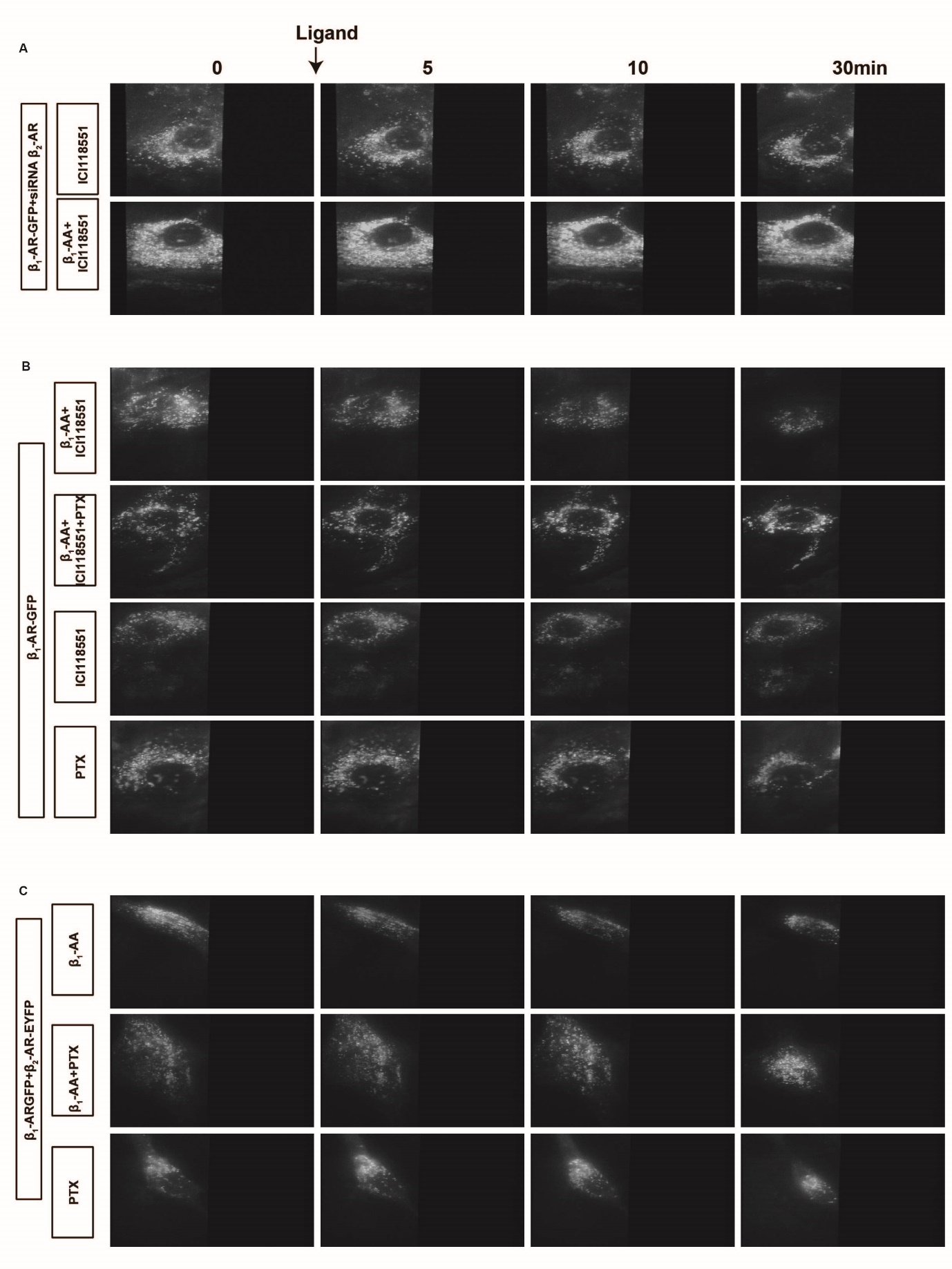
**

**Figure S24
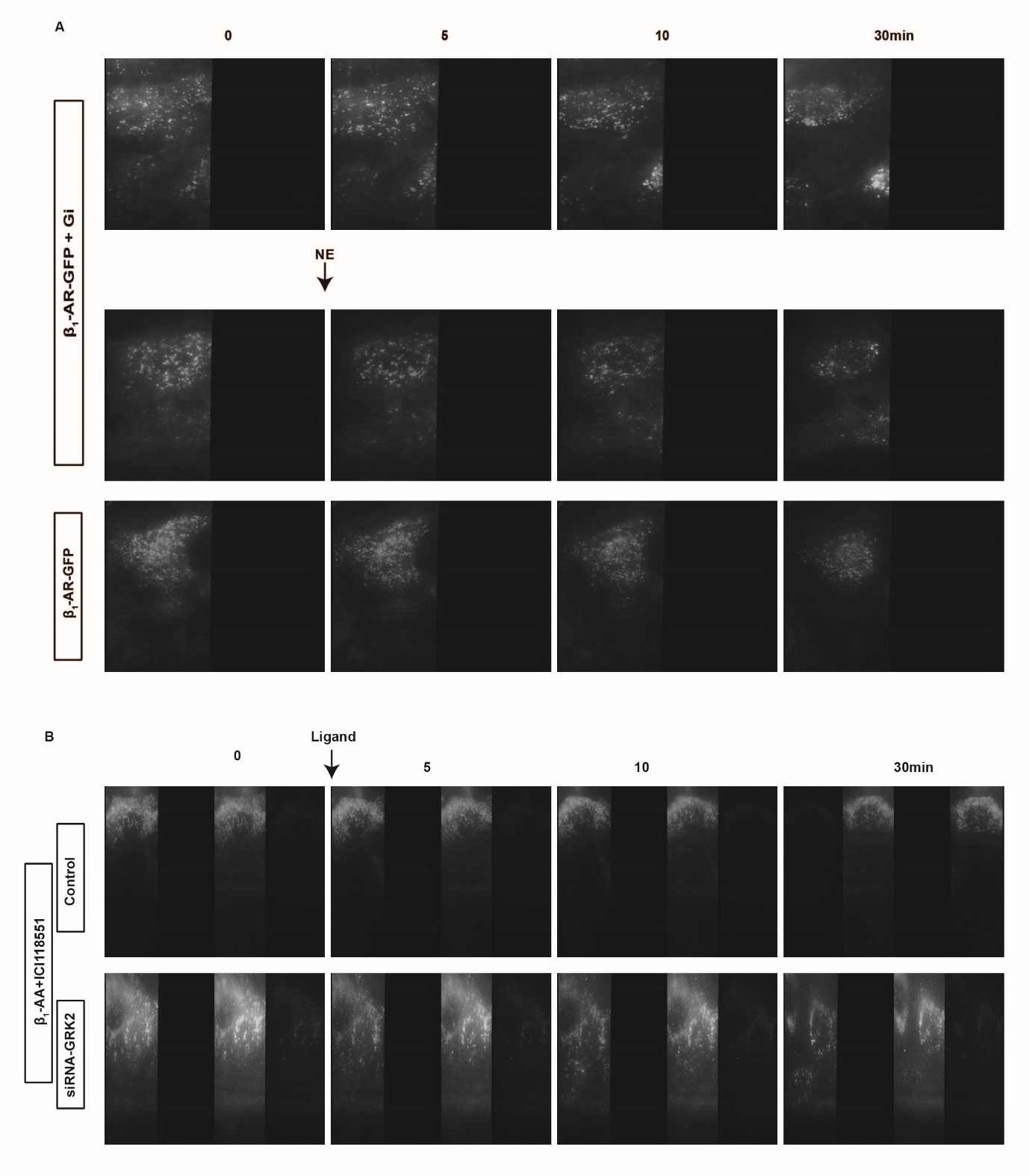
**

**Table S1: Clinical characteristics of selected patients with heart failure**

|  | β_1_-AA-positive patients | β_1_-AA-negative patients |
| --- | --- | --- |
| Sample number | 13 | 12 |
| β_1_-AA OD^1^ | 1.761±0.246 * | 1.005±0.145 |
| LVEF(%) | 53.462±8.643 | 55.75±7.166 |
| LVEDD(mm) | 12.231±4.117 | 12.167±3.555 |
| Systolic pressure | 126.846±13.427 | 126.417±13.847 |
| Diastolic pressure | 86.461±10.180 | 84.083±15.102 |
| Pathogeny | CHD | |
| NYHA | III-IV | |
| Age | 63±10.024 | 58.083±5.446 |
| Sex | male | |

**P* vs β_1_-AA negative patients, *P*<0.05 (unpaired t test).

**Table S2: Antibodies information in our research**

| **Antibody** | **Species** | **Catalog** | **Source** | **Western**  **blot** | **Immunoprecipitation** |
| --- | --- | --- | --- | --- | --- |
| β_1_-AR | rabbit | ab3442 | Abcam | 1:1000 |  |
| GAPDH | rabbit | ab128915 | Abcam | 1:1000 |  |
| Gαi | mouse | 26003 | NewEast  Biosciences | 1:1000 |  |
| active Gai-GTP | mouse | 26901 | NewEast  Biosciences |  | 1 μg/mg  total protein |
| β_2_-AR | rabbit | 182136 | Abcam | 1:1000 |  |
| β-arrestin1 | rabbit | bs-20232R | Bioss | 1:1000 |  |
| β-arrestin2 | rabbit | bs-1332R | Bioss | 1:1000 |  |
| Phospho-(Ser/Thr) Phe antibody | rabbit | 9631 | Cell Signaling | 1:1000 |  |
| GRK2 | rabbit | A4443 | ABclonal | 1:1000 |  |
| Phosphor-GRK2 (Ser29) | rabbit | bs-16683R | Bioss | 1:1000 |  |
| Phosphor-PKAR2 (Ser99) | rabbit | bs-4022R | Bioss | 1:1000 |  |
| PKA R2 | rabbit | bs-3963R | Bioss | 1:1000 |  |
| GFP | mouse | 66002-1-Ig | proteintech |  | 1μl for 200μg protein |

**Figure S1. The effect evaluation of different drugs acting alone**

**A**, Phe (10^-5^ M), Met (10^-5^ M) and β_1_-AR-ECII (10^-5^ M) had no effect on beating frequency of NRCMs. n=4. **B**, The change of Ca^2+^ in HL-1 cells was not affected by β_1_-AR-ECII (10^-5^ M). n=3.

**Figure S2. Successful transient transfection of β_1_-AR-GFP into HL-1 cells**

**A**, The expression of β_1_-AR-GFP in HL-1 cells was detected by fluorescence microscope. **B**, Western blot showed that the expression of β_1_-AR-GFP protein in HL-1 cells increased 48 hours after transfection. Unpaired t test was used to compare the transfected group and non-transfected control group. n=3. **P* vs Control, *P*<0.05.

**Figure S3. The weak recruitment of β_1_-AR with β-arrestin1 or β-arrestin2 under the treatment of β_1_-AA.**

**A**, The β_1_-AR-EYFP transfected into HEK293 cells was observed by fluorescence microscope. **B, C, D,** After 48 hours of transfection, Western blot showed the expressions of β_1_-AR-EYFP, β-arr1-Rluc (β-arrestin1-Rluc) and β-arr2-Rluc (β-arrestin2-Rluc) proteins in HEK293 cells. n=3. **P* vs Control, *P*<0.05 (unpaired t test). **E**, The schematic diagram of BRET. **F** and **G**, BRET was used to detect the concentration dependence of β_1_-AR-EYFP and β-arr1-Rluc or β-arr2-Rluc recruitment in HEK293 cells after incubation with different ligands for 20 minutes. Along the increase of NE dose, β_1_-AR-EYFP showed gradually increased recruitment with β-arr2-Rluc (**G**) and a trend of recruitment with β-arr1-Rluc (**F**). However, neither β-arr1-Rluc nor β-arr2-Rluc could recruit to β_1_-AR-EYFP under the β_1_-AA action, even if the concentration reached 10^-5^ M. n=8-9. **P* vs β_1_-AA, *P*<0.05 (2-way ANOVA with Bonferroni post hoc test).

**Figure S4. The β_1_-AR-GFP and β-arrestin1-RFP/β-arrestin2-RFP were successfully transiently transfected into HL-1 cells.**

**A**, The expression of β_1_-AR-GFP, β-arrestin1-RFP (β-arr1-RFP) and β-arrestin2-RFP (β-arr2-RFP) proteins in the HL-1 cells were observed under fluorescence microscope. **B**, After transfected for 48 hours, increased expressions of β_1_-AR-GFP, β-arr1-RFP, and β-arr2-RFP proteins in HL-1 cells were confirmed by Western blot. n=3. **P* vs Control (non-transfected group), *P*<0.05 (unpaired t test).

**Figure S5. Successful interference of endogenous β_2_-AR in HL-1 cells.**

**A** and **B**, the expression of β_2_-AR protein in HL-1 cells was significantly decreased after 72 hours of transfection with siRNA β_2_-AR as shown by Western blot. The β_2_-AR expressions in β_2_-AR siRNA and control siRNA groups were compared by unpaired t test. n=3. **P* vs Control, *P*<0.05.

**Figure S6. The transient transfection of β_2_-AR-EYFP into HL-1 cells.**

**A** and **B**, As exhibited by fluorescence microscope and Western blot, the expression of β_2_-AR-EYFP protein in HL-1 cells was markedly up-regulated after 42 hours of transfection. n=3. **P* vs Control (non-transfected group), *P*<0.05 (unpaired t test).

**Figure S7. ICI118551 biasedly activated β_2_-AR/Gi pathway, which was inhibited by PTX.**

The active Gi was determined by immunoprecipitation experiment. **A** and **B**, when HL-1 cells were stimulated with 10^-5^-10^-9^ M ICI118551 for 5 minutes, Gi activation was detected, while 10^-6^-10^-9^ M ICI118551 had no significant effect on it. n=3. **P* vs Control, *P*<0.05 (one-way ANOVA with Dunnett test). **C** and **D**, PTX (1.5 μg/ml) treated HL-1 cells for 13 hours and then stimulated with ICI118551 (10^-5^ M) for 5 minutes to detect Gi activation. PTX could significantly inhibit Gi activation caused by ICI118551. n=3. **P* vs Control, *P*<0.05, #*P* vs ICI118551, *P*<0.05 (one-way ANOVA with Dunnett test).

**Figure S8. Overexpression of Gi did not enhance endocytosis of β_1_-AR stimulated by NE in HL-1cell.**

The endocytosis of β_1_-AR was tested by TIRF in HL-1 cells overexpressing β_1_-AR-GFP and Gi. **A** is the typical pictures. **B** is the statistical charts of the effects of NE stimuli on β_1_-AR-GFP endocytosis. Scale bar=10 μm. Data were analyzed by 2-way ANOVA, followed by a Bonferroni test. n=3-4/group. *P vs β_1_-AA, P<0.05.

**Figure S9. After interference with GRK2 expression, the endocytosis promoting effect of ICI118551 disappeared.**

**A** and **B**: 72 hours after siRNA-GRK2 interfered with HL-1 cells, the expression of GRK2 in cells detected by Western blot showed that endogenous GRK2 was significantly reduced. n=3. **P* vs Control, *P*<0.05 (unpaired t test). **C** and **D**: ICI118551 (10^-5^ M) pretreated HL-1 cells with reduced GRK2 expression for 5 minutes and then given β_1_-AA stimulation, β_1_-AR-GFP endocytosis was significantly reduced. Scale bar=10 μm. Data were analyzed by 2-way ANOVA, followed by Bonferroni test. n=3/group. **P* vs Control, P<0.05.

**Figure S10. ICI118551 played a cardioprotective role in β_1_-AA-positive mice.**

**A**: Flow chart of β_1_-AA-positive mouse establishment and ICI118551 therapy. The mice were divided into 4 groups: saline group, β_1_-AA, β_1_-AA plus ICI118551 group, and ICI118551 group. **B** and **C**: M-mode echocardiographic study of cardiac function of mice in different groups. Unpaired t-tests were used to compare the changes in cardiac function indexes before and after administration. **P* vs 0M, n=4-5/group, *P*<0.05. **D**: HE staining showed changes in mouse myocardial structure (stereo picture: 2×, micrograph picture: 70×).

**Figure S11. The level of β_1_-AA in Balb/c mice before and after administration.**

After 2 months of injection, the levels of β_1_-AA in mice of β_1_-AA and β_1_-AA plus ICI118551 groups were significantly increased determined by enzyme linked immunosorbent assay. n=4-5. **P* vs 0M, *P*<0.05 (unpaired t test).

**Figure S12. The protein expression detection in HL-1 cells and NRCMs**

A: β_1_-AR, β_2_-AR, β-arrestin1, β-arrestin2 were expressed in HL-1 cells and NRCMs. HEK293 cells were used as control

B: α_1_-AR is not expressed in HL-1 cells. NRCMs and HEK293 cells were used as control.

**Figure S13. β_1_-AA slightly increased the phosphorylation level of β_1_-AR-GFP.**

**A** and **B**: Immunoprecipitation was used to detect the phosphorylation of β_1_-AR-GFP. Compared with NE (10^-5^ M) group, the phosphorylation level of β_1_-AR-GFP increased slightly when stimulated by β_1_-AA (10^-7^ M); and compared with β_1_-AA alone, the phosphorylation of β_1_-AR-GFP after ICI118551 (10^-5^ M) and β_1_-AA co-stimulation was slightly decreased. n=3/group.

**Figure S14. Raw data of the original unedited gel images.**

**A1** and **A2**: Unedited western blot figures for Figure S2B. **B1** and **B2:** Unedited western blot figures for Figure S3B. **C1** and **C2:** Unedited western blot figures for Figure S3C. **D1** and **D2:** Unedited western blot figures for Figure S3D.

**Figure S15. Raw data of the original unedited gel images.**

**A1** and **A2**: Unedited western blot figures for Figure S4B β_1_-AR-GFP overexpression. **B1** and **B2:** Unedited western blot figures for Figure S4B β-arrestin1-RFP overexpression. **C1** and **C2:** Unedited western blot figures for Figure S4B β-arrestin2-RFP overexpression. **D1** and **D2:** Unedited western blot figures for Figure S5A.

**Figure S16. Raw data of the original unedited gel images.**

**A1** and **A2**: Unedited western blot figures for Figure S6A. **B1**-**B3:** Unedited western blot figures for Figure S7A.

**Figure S17. Raw data of the original unedited gel images.**

**A1** and **A2**: Unedited western blot figures for Figure S7C. **B1** and **B2:** Unedited western blot figures for Figure 6A.

**Figure S18. Raw data of the original unedited gel images.**

**A1** and **A2**: Unedited western blot figures for Figure 6B. **B1** and **B2:** Unedited western blot figures for Figure S9A. **C1-D4:** Unedited western blot figures for Figure S12A. **E1-E2:** Unedited western blot figures for Figure S12B.

**Figure S19. Raw data of the original unedited gel images.**

**A1** and **A2**: Unedited western blot figures for Figure S13A.

**Figure S20. Raw data of the original unedited TIRF images.**

Unedited TIRF figures for Figure 1E.

**Figure S21. Raw data of the original unedited TIRF images.**

**A**: Unedited TIRF figures for Figure 2C. **B**: Unedited TIRF figures for Figure 2E.

**Figure S22. Raw data of the original unedited TIRF images.**

**A**: Unedited TIRF figures for Figure 3D. **B**: Unedited TIRF figures for Figure 3F. **C**: Unedited TIRF figures for Figure 4D.

**Figure S23. Raw data of the original unedited TIRF images.**

**A**: Unedited TIRF figures for Figure 5B. **B**: Unedited TIRF figures for Figure 5E. **C**: Unedited TIRF figures for Figure 5H.

**Figure S24. Raw data of the original unedited TIRF images.**

**A**: Unedited TIRF figures for Figure S8A. **B**: Unedited TIRF figures for Figure S9C.

**Legends for Video files**

**Videos S1:** Intracellular Ca^2+^ fluorescence intensity of HL-1 cells increased temporarily under NE stimulation.

**Videos S2:** The fluorescence intensity of Ca^2+^ in HL-1 cells continuously increased after being treated by β_1_-AA.

**Table S3:** The original data of figure 1A.

**Table S4:** The original data of figure 1B.

**Table S5:** The original data of figure 1C.

**Table S6:** The original data of figure 1F.

**Table S7:** The original data of figure S1A.

**Table S8:** The original data of figure S1B.

**Table S9:** The original data of figure 2A.

**Table S10:** The original data of figure 2B.

**Table S11:** The original data of figure 2D.

**Table S12:** The original data of figure 2F.

**Table S13**: The original data of figure 2G.

**Table S14**: The original data of figure 2H.

**Table S15**: The original data of figure S3F.

**Table S16**: The original data of figure S3G.

**Table S17**: The original data of figure 3A.

**Table S18**: The original data of figure 3B.

**Table S19**: The original data of figure 3C.

**Table S20**: The original data of figure 3E.

**Table S21**: The original data of figure 3G.

**Table S22**: The original data of figure 3H.

**Table S23**: The original data of figure 4A.

**Table S24**: The original data of figure 4B.

**Table S25**: The original data of figure 4C.

**Table S26**: The original data of figure 4E.

**Table S27**: The original data of figure 5A.

**Table S28**: The original data of figure 5C.

**Table S29**: The original data of figure 5D.

**Table S30**: The original data of figure 5F.

**Table S31**: The original data of figure 5G.

**Table S32**: The original data of figure 5I.

**Table S33**: The original data of figure S8B.

**Table S34**: The original data of figure S9D.

**Table S35**: The original data of figure S11.

**Table S36**: The original data of figure S10C.
