# Supplementary material for: Biased activation of β2-AR/Gi/GRK2 signal pathway attenuated β1-AR sustained activation induced by β1-adrenergic receptor autoantibody
Source: Cell Death Discov. 2021 Nov 8;7:340. doi: 10.1038/s41420-021-00735-2 (PMC8576015; doi:10.1038/s41420-021-00735-2)
Supplement: Supplementary file 8 — Table S7 [file 41420_2021_735_MOESM8_ESM.pdf]

Time (min)	Phe				β1-AR-EC II			
0	0	0	0	0	0	0	0	0
5	-8	-12	-20	4	-8	-4	-4	
15	4	12	-12	0	-20	-16	12	-12
30	12	16	-4	16	-8	-16	-4	20
45	0	4	-4	0	4	-16	32	4
60	20	4	20	16	-4	-8	20	24
75	12	0	28	0	4	-16	24	-16
90	4	0	16	4	0	4	20	16

MET			
0	0	0	0
-4	0	-8	-8
-8	0	-8	-32
-8	8	-32	0
0	0	16	12
0	16	-24	-4
4	8	-24	0
0	8	24	-8
