# Supplementary material for: Biased activation of β2-AR/Gi/GRK2 signal pathway attenuated β1-AR sustained activation induced by β1-adrenergic receptor autoantibody
Source: Cell Death Discov. 2021 Nov 8;7:340. doi: 10.1038/s41420-021-00735-2 (PMC8576015; doi:10.1038/s41420-021-00735-2)
Supplement: Supplementary file 9 — Table S8 [file 41420_2021_735_MOESM9_ESM.pdf]

Time (s)	Control	β_1-AR ECⅡ							
0	1	1							
4.21	0.990776	0.9817771							
8.29	0.983386	0.9644778							
12.52	0.978854	0.9474228							
16.76	0.973003	0.9344751							
20.77	0.968896								
24.99	0.965664	0.9329743							
28.99	0.962924	0.9158522							
33.13	0.953378	0.91619							
37.18	0.937647	0.9144177							
41.23	0.942138	0.9002399							
45.56	0.938522	0.8850805							
49.43	0.935871	0.879706							
53.68	0.933882	0.8635727							
61.86	0.924272	0.8350919							
66.17	0.916507	0.8349081							
70.16	0.917556								
74.38	0.903008	0.8242443							
78.36	0.907635	0.8214465							
82.28	0.903159								
86.36	0.904773	0.8033045							
90.61	0.897149	0.7911643							
94.57	0.894711	0.7835376							
98.58	0.893315	0.7771819							
102.94	0.887606	0.7783135							
107.06	0.887977								
111.36	0.887284	0.7757888							
115.75	0.887668	0.7735451							
119.85	0.888377	0.7697543							
124.09	0.880726								
128.2	0.880094	0.7691106							
132.58	0.883463	0.7725396							
136.9	0.889314	0.7565085							
141.06	0.880306	0.7485659							
145.22	0.88359	0.7556016							
149.35	0.884934								
153.55	0.889871	0.7438335							
157.75	0.891983	0.7456228							
161.96	0.880397	0.7473225							
166.15	0.875607	0.747779							
170.51	0.87624	0.7429385							
174.61		0.7369665							
178.81	0.870845	0.7369645							
182.97	0.865049								
187.14	0.866792	0.7365392							
191.37	0.871523	0.7340791							
195.51	0.870107	0.7350782							
199.51	0.862671	0.7297814							
203.63	0.863034								
		0.7233376							
207.89	0.86053								
211.87	0.858247	0.7149077							
216.17	0.861151	0.7244095							
220.54	0.850772	0.7227687							
224.51	0.857142								
228.84	0.851726	0.727787							

233.16	0.85669							
237.26	0.865113	0.7220764						
241.48	0.866677	0.7132997						
245.73	0.862254							
250.12	0.866095	0.7179812						
254.33	0.863443	0.7150089						
258.6	0.861063	0.7176817						
262.61	0.868764	0.7204509						
266.81	0.862012	0.716632						
271.02	0.862202	0.7118222						
275.42	0.862851	0.7129182						
279.71	0.859122	0.7100285						
284.03	0.852664							
288.3	0.847502	0.7002669						
292.49	0.850658	0.7014548						
296.65	0.850613	0.6992416						

